# Quantification of soluble epoxide hydrolase inhibitors in experimental and clinical samples using the nanobody-based ELISA

**DOI:** 10.1016/j.jpha.2023.05.006

**Published:** 2023-05-16

**Authors:** Huiyi Yang, Meng Qi, Qiyi He, Sung Hee Hwang, Jun Yang, Mark McCoy, Christophe Morisseau, Suqing Zhao, Bruce D. Hammock

**Affiliations:** a Department of Pharmaceutical Engineering, School of Biomedical and Pharmaceutical Sciences, Guangdong University of Technology, Guangzhou, China; b Department of Entomology and Nematology and UCD Comprehensive Cancer Center, University of California, Davis, CA 95616, USA; c Langfang Normal University, Langfang, Hebei, 065000, China

**Keywords:** Nanobody, Immunoassay, Soluble epoxide hydrolase inhibitors, Metabolites, Small molecules

## Abstract

To ensure proper dosage of a drug, analytical quantification of it in biofluid is necessary. Liquid chromatography mass spectrometry (LC-MS) is the conventional method of choice as it permits accurate identification and quantification. However, it requires expensive instrumentation and is not appropriate for bedside use. Using soluble epoxide hydrolase (sEH) inhibitors (EC5026 and TPPU) as examples, we report development of a nanobody-based enzyme-linked immunosorbent assay (ELISA) for such small molecules and its use to accurately quantify the drug chemicals in human samples. Under optimized conditions, two nanobody-based ELISAs were successfully established for EC5026 and TPPU with low limits of detection of 0.085 ng/mL and 0.31 ng/mL, respectively, and two order of magnitude linear ranges with high precision and accuracy. The assay was designed to detect parent and two biologically active metabolites in the investigation of a new drug candidate EC5026. In addition, the ELISAs displayed excellent correlation with LC-MS analysis and evaluation of inhibitory potency. The results indicate that nanobody-based ELISA methods can efficiently analyze drug like compounds. These methods could be easily implemented by the bedside, in the field in remote areas or in veterinary practice. This work illustrates that nanobody based assays offer alternative and supplementary analytical tools to mass spectrometry for monitoring small molecule medicines during clinical development and therapy. Attributes of nanobody based pharmaceutical assays are discussed.

## Highlights

• High-affinity nanobodies against soluble epoxide hydrolase (sEH) inhibitors were reported.
• Rapid and sensitive nanobody-based ELISA to quantify sEH inhibitors was developed.
• The new nanobody–based ELISA correlated well with LC-MS analysis for the detection of sEH inhibitors.
• The new ELISA was used to analyze urine samples of human dosed with EC5026.
• Future role of nanobodies in pharmaceutical discovery and application was discussed.

## Graphical abstract

**Figure:**
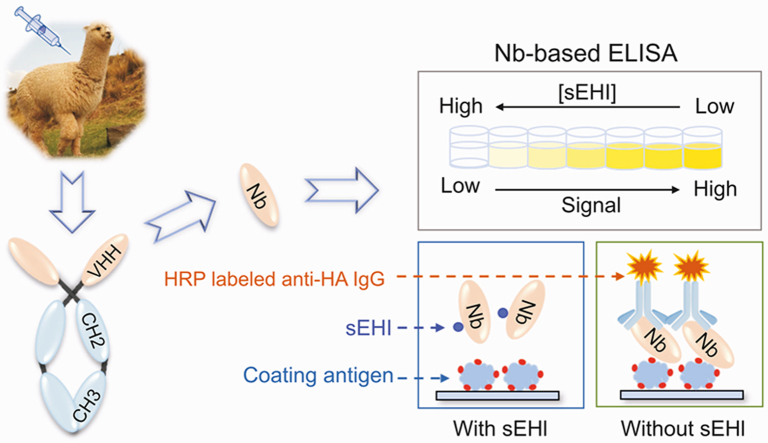

## 1 Introduction

The soluble epoxide hydrolase (sEH) enzyme is a promising target for pharmaceutical development. Epoxy fatty acids (EpFAs) are important endogenous lipid mediators that resolve inflammation in part by modulating nuclear factor-kappa-B (NF-κB) signaling and the endoplasmic reticulum stress pathway. The sEH in mammals is a 125 kDa antiparallel homodimer that is widely distributed and rapidly inactivates epoxy fatty acids to their 1,2-diols [[1], [2], [3], [4]]. Inhibition of sEH increases levels of EpFAs and is a promising approach for the treatment of a variety of diseases, such as inflammation [5], hypertension [6], neuropathic pain [7], diabetes [8], and central nervous system disorders [9]. Therefore, a series of sEH inhibitors (sEHI) inhibitors have been developed to stabilize EpFAs. Among these sEHI, EC5026 has been successfully moved through human phase 1a trials with no observed drug-related adverse events [10]. Another sEHI 1-trifluoromethoxyphenyl-3-(1-propionylpiperidin-4-yl)urea (TPPU) is widely used in research due to its excellent pharmacokinetic profile, target potency, and in vivo biological activity with no known off-target effects [11].

Methods based on liquid chromatography-tandem mass spectrometry (LC-MS/MS) have been developed to detect sEHIs [12]. Such methods are expected for registration by US Food and Drug Administration (US FDA) and excellent for the identification and sensitive quantification of sEHIs and their metabolites in various kinds of samples as long as time and cost are not primarily concerns. However, these sequential methods require expensive instruments, highly trained personnel, and long time periods. The instruments may not be available in smaller facilities. As the sEHI are increasingly used as biochemical probes, a variety of laboratories need to determine the levels of sEHI to interpret their physiological data. The enzyme-linked immunosorbent assays (ELISAs) can be run in laboratories or field situations with improvements in cost-effectiveness, convenience, high sensitivity and reliability [[13], [14], [15]]. Therefore, using sEHI (EC5026 and TPPU) as model compounds, here an ELISA for such small molecules was developed and was used to accurately quantify the chemical in human samples.

The key to an outstanding ELISA is obtaining antibodies with high affinity, sensitivity, batch-to-batch consistency, and specificity. However, industry and academia have been confronted with reproducibility issues of antibodies due to limited supply and large lot-to-lot variability of polyclonal antibodies (pAb) or the tendency of monoclonal antibodies (mAb) hybridoma cells to die or be lost while recovering from storage [16]. Nanobodies (Nb), also known as variable heavy chain antibodies (VHH), are recombinant antibodies with a single variable domain derived from heavy-chain-only antibodies in camelids and sharks, which can be easily sequenced and resynthesized to solve the reproducibility issues [17,18]. Compared with traditional pAb or mAb, Nb have attracted increasing attention because of their small size, genetic operability, monoclonal nature, high thermal stability, excellent solubility, easy clonal storage, and ease of expression in different expression platforms [19,20]. Therefore, it is not surprising that Nb are gradually replacing traditional IgG-based antibodies in various immunological techniques, especially in ELISA quantification methods [21]. However, to date nanobodies have not been found wide use in pharmaceuticals analysis [22]. In particular, Nb-based immunoassays for sEHIs (EC5026 and its bioactive metabolites, and TPPU) have not been reported.

Toward this goal, in this work, nanobodies with high affinity to the sEHI EC5026 and TPPU were obtained and used to develop sensitive indirect competitive ELISAs. Precision and accuracy of the ELISAs were determined, and the structure activity relationship of the ELISAs was studied and validation was performed. This study outlines the techniques of the preparation of nanobodies for sEHIs from the hapten design through assay development and illustrates the advantages and limitations of nanobody technology.

## 2 Materials and methods

### 2.1 Chemicals and materials

EC5026, TPPU, and compounds used for the investigation of cross-reactivity (CR) were synthesized and characterized in previous reports [11,[23], [24], [25], [26]]. 3-fluoro-4-(trifluoromethoxy)aniline (compound 14) was purchased from COMBI BLOCKS Inc. (San Diego, CA, USA). 4-(trifluoromethoxy)aniline (compound 15) was purchased from AK Scientific Inc. (Union City, CA, USA). Freund's adjuvant, thyroglobulin (Thy), bovine serum albumin (BSA), ovalbumin (OVA), 3,3′,5,5′-tetramethylbenzidine (TMB), polyethylene glycol 8000 (PEG 8000), isopropyl-β-d-thiogalactopyranoside (IPTG), dicyclohexyl carbodiimide (DCC), and *N*-hydroxysuccinimide (NHS) were purchased from Sigma-Aldrich Chemical Co. (St. Louis, MO, USA). Electrocompetent ER2738 cells from *E. coli* were acquired from Lucigen Corporation (catalog #60522−1; Middleton, WI, USA). M13KO7 helper phage (catalog #N0315S) and *Sfi*I (catalog #R0123S) were purchased from New England Biolabs (Ipswich, MA, USA). HRP labeled anti-HA tag antibody conjugate (HRP-anti-HA) was purchased from Roche (catalog #34071100; Basel, Switzerland). Bacterial protein extraction reagent (B-PER), HisPur Ni-NTA resin, high-binding microplates (catalog #442404) and fetal bovine serum (FBS, catalog #16140089) were purchased from Thermo Fisher Scientific (Rockford, IL, USA). The human urine sample used for spike-recovery study was collected from a volunteer, and the human urine samples collected from the human clinical trial (NCT04228302) were used for ELISA validation. The human clinical trial (NCT04228302) was conducted following International Council for Harmonisation Good Clinical Practice guidelines and ethical principles that have their origin in the Declaration of Helsinki. The study protocol was reviewed and approved by an investigational review board before the study began, and informed consent was obtained from each subject before entering the study. DNA sequencing was conducted by the UC Davis DNA Sequencing Facility. Phosphate-buffered saline (PBS, 10 mM, pH 7.4) contains 8.0 g NaCl, 2.9 g Na_2_HPO_4_ and 0.2 g NaH_2_PO_4_.

### 2.2 Synthesis of haptens and preparation of immunogens and coating antigens

Synthesis of haptens, immunogens and coating antigens are detailed in the Supplementary data. The starting materials 1-(3-fluoro-4-(trifluoromethoxy)phenyl)-3-(piperidin-4-yl)urea (compound **11**) and 1-(piperidin-4-yl)-3-(4-trifluoromethoxy)phenyl) urea (compound **12**) were prepared by literature methods [10,11]. 1-Thy and 2-Thy were the immunogens for EC5026, and 4-Thy and 5-Thy were the immunogens for TPPU. 1-BSA, 1-OVA, 2-BSA, 2-OVA, 3-BSA, and 3-OVA were evaluated as coating antigens (cAgs) for EC5026, while 4-BSA, 4-OVA, 5-BSA, 5-OVA, 6-BSA and 6-OVA were served as cAgs for TPPU.

### 2.3 Construction of phage display library and screening of the nanobody

Construction of phage display library and screening of the nanobodies are detailed in the Supplementary data. The conditions for each round of screening are listed in Table S1. After three rounds of panning was accomplished, several clones were randomly selected from the output plates and the binding affinity to EC5026 or TPPU (1 μg/mL) was identified by indirect competitive phage ELISA. The plasmids of positive clones were extracted and sequenced for further analysis.

### 2.4 Expression, purification, and characterization of sEHI Nbs

Expression, purification, and characterization of sEHI Nbs are detailed in the Supplementary data.

### 2.5 Evaluating performance of Nb-based immunoassay

The Nb-based ELISAs for EC5026 and TPPU were performed as followed: (a) 100 μL of cAg (2-BSA for EC5026, or 6-OVA for TPPU, 0.5 μg/mL) in carbonate buffer (10 mM, pH 9.6) was coated in the 96-well microplate and incubated at 4 °C overnight; (b) 250 μL of skim milk powder in PBS (3%) was transferred and shaken at 25 °C for 60 min after the microplate was washed with 0.05% PBST (PBS containing 0.05% Tween-20); and (c) 50 μL of Nb in PBS (125 ng/mL of Nb-EC03, or 7.8 ng/mL of Nb-TP02) was incubated together with 50 μL of sEHI (EC5026 or TPPU) or their analogs in solutions ranging from 0 to 1000 ng/mL and shaken for 60 min, followed by being washed with 0.05% PBST; (d) 100 μL of HRP-anti-HA was introduced, incubated and shaken for another 60 min; (e) after the final washing step, 100 μL of the TMB substrate solution was added and incubated for 15 min, followed by being stopped by 50 μL of H_2_SO_4_ solution (1.0 M). The limit of detection (LOD) of Nb-based ELISAs for detecting the concentrations of the sEHI was defined as the concentration of sEHI that provided a 10% inhibition rate (IC_10_ value). The linear range was established between the IC_20_ value and the IC_80_ value. CR was calculated based on the equation: CR% = [IC_50_ (EC5026 or TPPU)/IC_50_ (analogs)] × 100%.

### 2.6 Clinical sample analysis via Nb-based ELISA and LC-MS

To assess the feasibility of Nb-based ELISAs for EC5026 and TPPU, the human urine and FBS were used as the matrix for precision and accuracy determination. The precision is shown as coefficient variation (CV%). The accuracy is expressed by the recovery, and the recovery is calculated as (Found concentration/Spiked concentration) × 100%. The standard solutions of EC5026 or TPPU were spiked blindly into human urine and FBS to obtain final concentrations of 1.25, 5 and 20 ng/mL, respectively. The experimental urine samples were collected from participants in the human clinical trial for EC5026, and the supernatant was collected by centrifugation (10,000 *g*, 5 min, 4 °C). The processed clinical samples were divided into two aliquots. One was diluted 10 times and then was detected by the Nb-based ELISA, and the other was for the LC-MS analysis directly in a bind fashion by different scientists.

## 3 Results and discussion

### 3.1 Design of haptens, immunogens and coating antigens

**Fig. 1:**
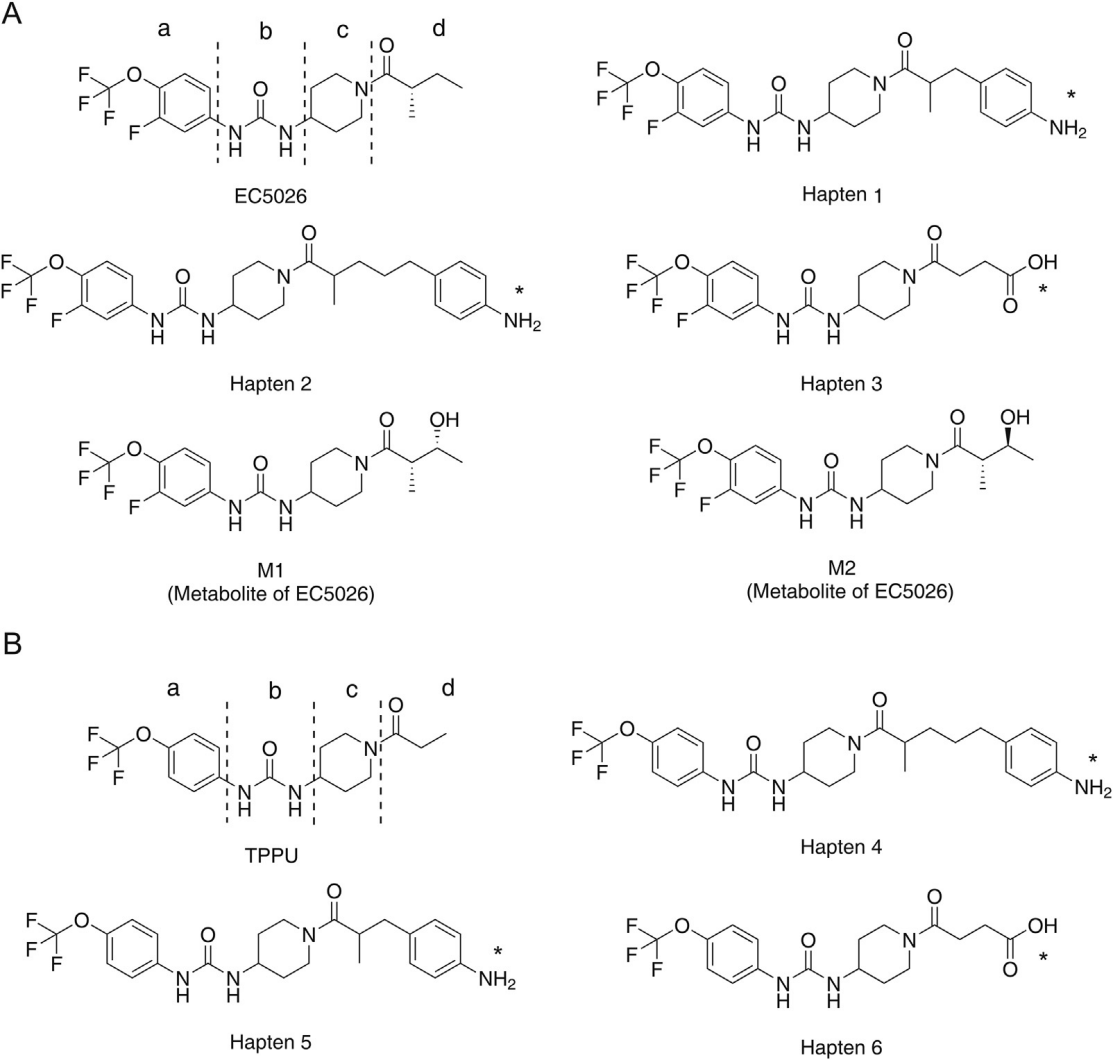
The structures of major compounds used in this study. (A) The structure of EC5026, its three haptens and major metabolites M1 and M2. (B) The structure of TPPU, and its three haptens. The structure of EC5026 and TPPU with the trifluoromethoxy phenyl groups (part a), the urea central pharmacophore (part b), the piperidine (part c) and tail (part d). ∗position of attachment to protein.

Haptens 1, 2 and 3 were designed for EC5026, while haptens 4, 5 and 6 were designed for TPPU (Fig. 1). EC5026, and its biologically active metabolites, M1 and M2, have the same chemical structures (parts a, b and c), except for a hydroxyl group (part d) (Fig. 1). Since sEHI are small molecules without immunogenic activity, they need to be coupled with the carrier protein to be immunogenic. Therefore, six haptens were designed on the right side of EC5026 and TPPU (part d) for conjugation with carrier proteins, so that nanobodies could simultaneously recognize both drug analytes and previously determined biologically active metabolites because antibodies are usually formed to recognize the furthest or distal side of the target molecule from the attachment site to the carrier protein [27]. No significant metabolites formed on the left side of EC5026 or TPPU were detected in humans or rodents or their microsomes. In this work, four hapten-protein conjugates (1-Thy, 2-Thy, 4-Thy, and 5-Thy) were chosen as immunogens to improve immunogenicity.

### 3.2 Isolation of sEHI Nbs

**Fig. 2:**
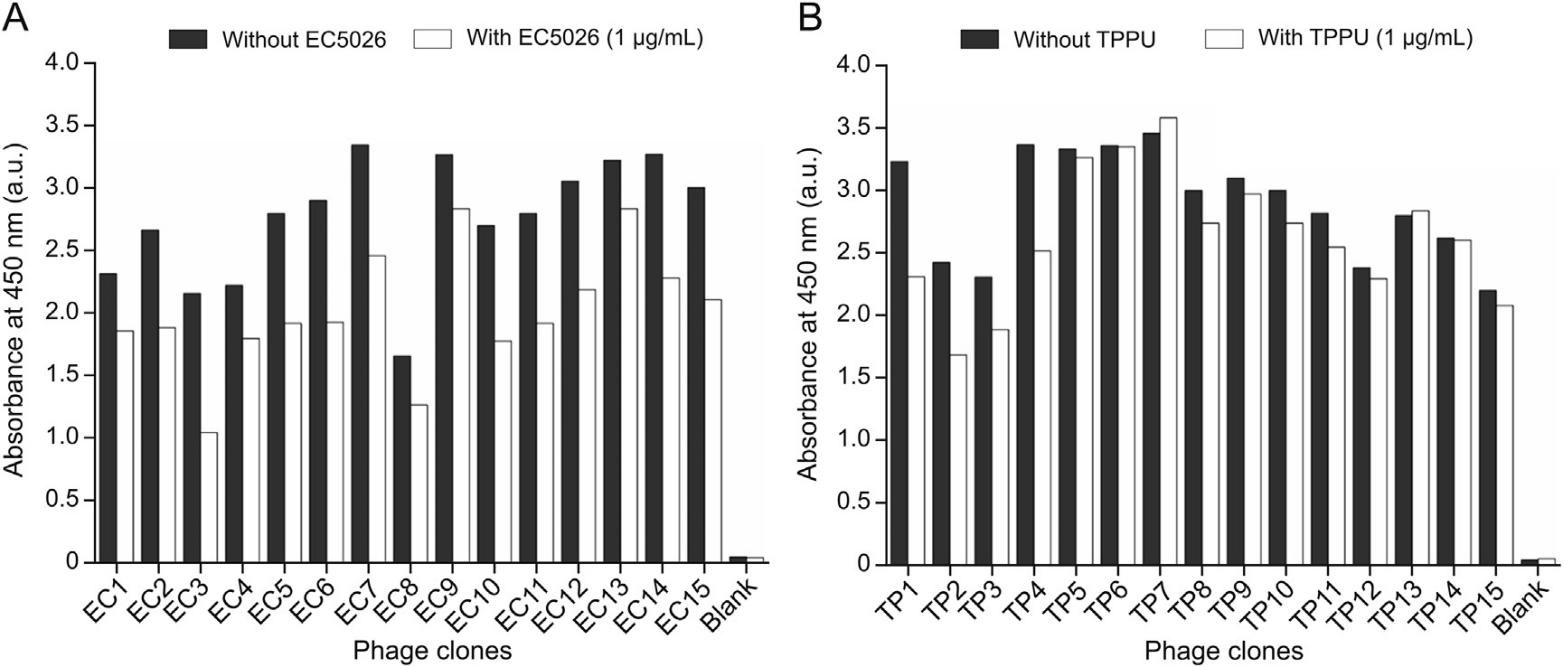
Phage clones binding to (A) EC5026 and (B) TPPU identified by phage enzyme-linked immunosorbent assay (ELISA). A large difference between the bars indicates a clone with high specific binding for the target analyte EC5026 or TPPU.

After the fifth immunization of the llama, the fresh blood was collected to construct the phage display library, which could be utilized for biopanning against EC5026 and TPPU, respectively. To increase chances of obtaining nanobody clones with high affinity to the analyte during the biopanning procedure, three cAgs of each inhibitor were used in the biopanning (1-BSA, 2-BSA or 3-BSA for EC5026, and 4-BSA, 5-BSA or 6-BSA for TPPU, respectively). After three rounds of biopanning, the titer of output phages increased, suggesting a significant enrichment of specific phage clones with the ability to bind to each of the cAgs. Subsequently, indirect competitive phage ELISA was used to evaluate the binding affinity of these enriched phages against analytes EC5026 and TPPU, respectively. As displayed in Fig. 2A, all the phage clones showed binding affinity for EC5026, resulting in lower absorbance and then part of them were selected randomly to be sequenced. As for TPPU, only four positive phage clones displayed great binding affinity (Fig. 2B). As shown in Figs. S1A and B, all phage clones showed the same length of amino acid sequences, but different framework regions (FR) and complementarity determining regions (CDR) after the sequence alignment analysis. Therefore, phage clones optimally targeting EC5026 and TPPU have two distinct amino acid sequences, respectively. Specifically, the sequences of phage clones EC02, EC04, EC06, and EC09 were virtually identical, which were different from that of phage clone EC03, while the sequences of phage clones TP01, TP02, and TP04 were almost identical, which were different from that of phage clone TP03. In this case, label EC stands for EC5026 and label TP stands for TPPU. The inhibition of these positive clones was rechecked by phage ELISA. As shown in Figs. S1C and D, phage clones EC03 and TP02 with the highest inhibition were chosen for transformation, expression, and purification of nanobodies against EC5026 and TPPU, respectively. Finally, Nbs, named Nb-EC03 for EC5026 and Nb-TP02 for TPPU, were obtained, respectively. Their molecular weight is approximately 18 kDa as determined by sodium dodecyl sulfate polyacrylamide gel electrophoresis (SDS-PAGE) (Fig. S2).

### 3.3 Characterization of sEHI Nbs

**Fig. 3:**
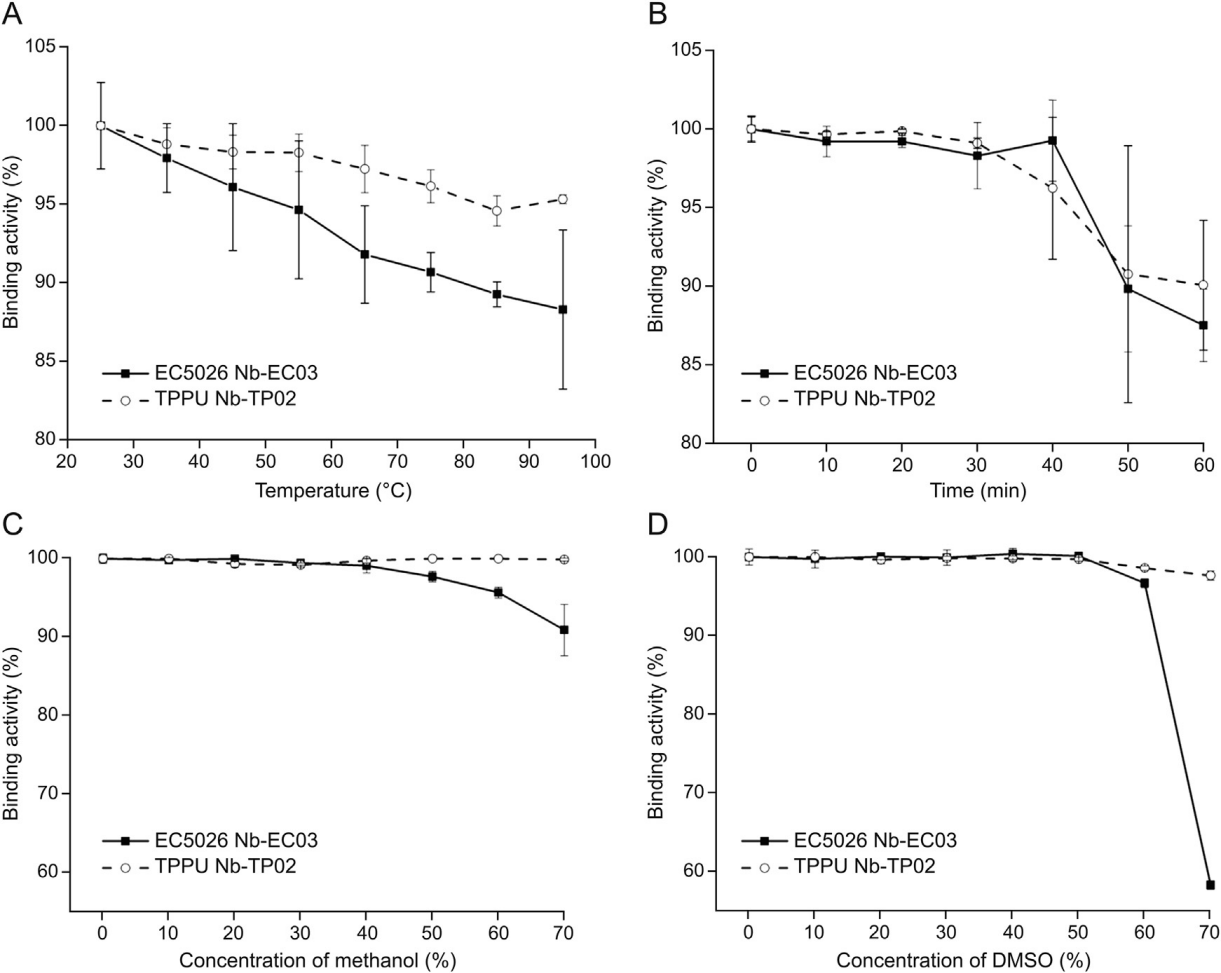
Thermostability and organic solvent tolerance of nanobodies determined by indirect enzyme-linked immunosorbent assay (ELISA). (A) The binding activity of Nb-EC03 and Nb-TP02 after being incubated at 25–95 °C for 5 min, respectively. (B) The binding activity of Nb-EC03 and Nb-TP02 after being incubated at 85 °C for 0–60 min, respectively. (C, D) The binding activity of Nb-EC03 and Nb-TP02 after being incubated by a series of final concentrations (0%–70% (*V/V*)) of methanol (C) and dimethylsulfoxide (DMSO) (D). Error bars denote the standard deviation (*n* = 3).

The thermostability investigation of Nb-EC03 and Nb-TP02 is displayed in Figs. 3A and B. Approximately 90% of the binding affinity of Nb-EC03 and Nb-TP02 was retained even after heating at 95 °C for 5 min. High stability of the binding affinity of Nb-EC03 and Nb-TP02 is thought to be due to disulfide bond shuffling and reversible refolding of the nanobody [[28], [29], [30]]. The excellent thermostability of Nb-EC03 and Nb-TP02 should increase shelf life, improve stability during the actual performance of the assay, and improve the stability of the nanobody in more advanced sensor formats. Furthermore, Nb-EC03 and Nb-TP02 exhibited outstanding tolerance to various concentrations of organic solvents (methanol and dimethylsulfoxide (DMSO)). Notably, the binding activity of Nb-EC03 and Nb-TP02 especially Nb-TP02, showed minimal change as methanol concentration increased (Fig. 3C). As displayed in Fig. 3D, the binding activity of Nb-EC03 dropped rapidly from 100% to 58.2% when the concentration of DMSO increased to 70%, whereas the binding activity of Nb-TP02 remained near 100%. The above results indicated that Nb-EC03 and Nb-TP02 displayed outstanding thermostability and solvent tolerance, suggesting their great potential for robust immunoassays and value in a range of sensor designs. In this work, parameters that show the most greatly influence on the interaction between the analyte and the nanobody were optimized. As for the parameters, such as washing buffer and time, that could have a lesser influence on the assay, general parameters were used here.

### 3.4 Selection of homologous and heterologous coating antigens

In this work, heterologous and homologous cAgs for EC5026 and TPPU were designed and synthesized, respectively. The haptens used in heterologous cAgs are similar to but different from those in immunogens, and the haptens used in homologous cAgs are identical to those in immunogens. Haptens 1, 2, 4, and 5 were used for immunogens, 1-Thy, 2-Thy, 4-Thy, and 5-Thy. Heterologous cAgs of EC5026 and TPPU were 3-BSA and 3-OVA, 6-BSA and 6-OVA, respectively. Others were homologous cAgs, 1-BSA, 1-OVA, 2-BSA, 2-OVA, 4-BSA, 4-OVA, 5-BSA, and 5-OVA. The concentration of cAg, Nb-EC03 and Nb-TP02 were optimized by checkerboard ELISA (Figs. S3 and S4). Taking cAg 3-BSA for example, as shown in Fig. S3C, absorbance did not increase with the increase of cAg concentration when the concentration of cAg exceeded 0.25 μg/mL. Thus, the optimal concentration of 3-BSA was 0.25 μg/mL. The optimal concentrations of all cAgs and nanobodies are listed in Table S2.

**Fig. 4:**
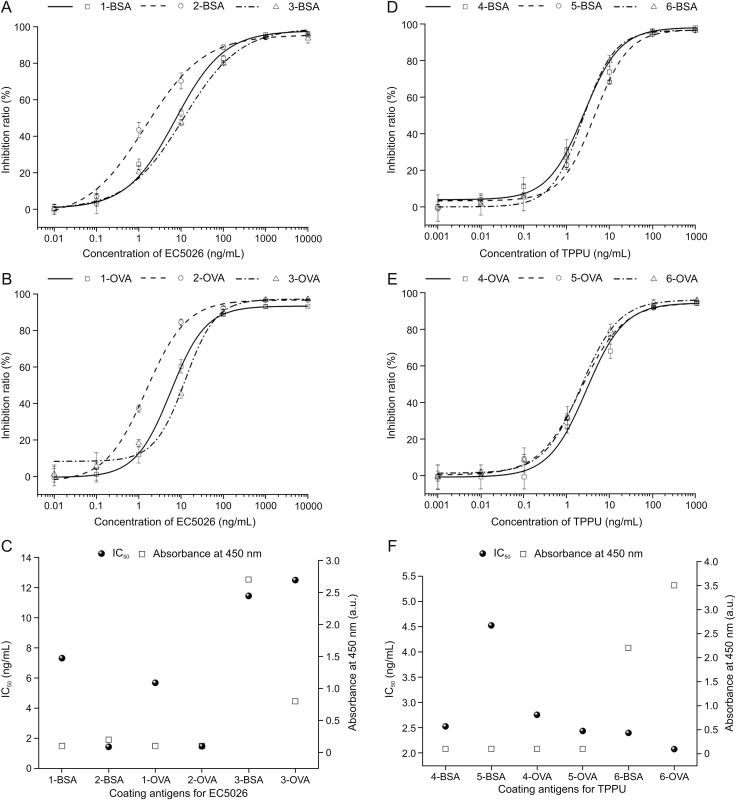
Standard curves of EC5026 and TPPU using all synthetic coating antigens determined by nanobodies (Nb)-based enzyme-linked immunosorbent assay (ELISA), and two indicators of these standard curves (IC_50_ and absorbance at 450 nm). (A) Standard curves of EC5026 with 1-BSA, 2-BSA and 3-BSA as coating antigens. (B) Standard curves of EC5026 with 1-OVA, 2-OVA and 3-OVA as coating antigens. (C) The IC_50_ and absorbance at 450 nm of Nb-based ELISA for EC5026 with all synthetic coating antigens. (D) Standard curves of TPPU with 4-BSA, 5-BSA and 6-BSA as coating antigens. (E) Standard curves of TPPU with 4-OVA, 5-OVA and 6-OVA as coating antigens. (F) The IC_50_ and absorbance at 450 nm of Nb-based ELISA for TPPU with all synthetic coating antigens. The error bar represents the standard deviation (*n* = 3). BSA: bovine serum albumin; OVA: ovalbumin.

Standard curves for EC5026 and TPPU were developed using all of the synthesized cAgs under the optimized conditions, and the effect of homologous and heterologous cAgs on sensitivity was investigated (Fig. 4). As shown in Fig. 4C and Table S3, the IC_50_ values of homologous cAgs, 1-BSA, 1-OVA, 2-BSA, 2-OVA, were at least 1.6-fold lower than those of heterologous cAgs, 3-BSA and 3-OVA. For the highest sensitivity, 2-BSA was chosen as the cAg for subsequent investigations with the Nb-based ELISA for EC5026. As exhibited in Fig. 4F and Table S4, the IC_50_ values of cAgs for the Nb-based ELISA for TPPU were similar except for 5-BSA. Regardless of EC5026 or TPPU sensing system, the signal strength of heterologous cAgs was higher than that of homologous cAgs, demonstrating that heterologous cAgs produced higher working titers of reagents. To obtain higher sensitivity and titer, 6-OVA was chosen for the following exploration of the TPPU immunoassay.

### 3.5 Optimization of experimental factors

To achieve an appropriate performance of Nb-based ELISAs, some experimental factors, such as the concentration of methanol, pH, and ionic strength of the sensing system, were explored. To determine an optimal parameter, three indicators with a higher maximum absorbance (*A*_max_), a lower IC_50_ value, and a higher ratio of *A*_max_/IC_50_ were selected for the higher sensitivity. The relationship among three indicators and the methanol concentration is demonstrated in Figs. S5A and B and S6A and B. The IC_50_ value increased with the concentration of methanol from 5% to 40%, and the highest ratio of *A*_max_/IC_50_ was at 5% methanol. As exhibited in Figs. S5C and D and S6C and D, the lowest IC_50_ value and the highest *A*_max_/IC_50_ value occurred at pH 7.4, suggesting Nb-based ELISAs for both EC5026 and TPPU performed well at pH 7.4. The assay was investigated with various concentrations of PBS solutions, ranging from 10 to 50 mM, and results are demonstrated in Figs. S5E and F and S6E and F. Similarly, the optimum ionic strength of PBS solutions was 10 mM with the highest *A*_max_/IC_50_ value. In summary, assays work well under a variety of conditions but, the best methanol concentration, pH, and ionic strength for Nb-based ELISAs for EC5026 and TPPU was 5%, 7.4, and 10 mM, respectively.

### 3.6 Analytical performance of Nb-based ELISA

**Fig. 5:**
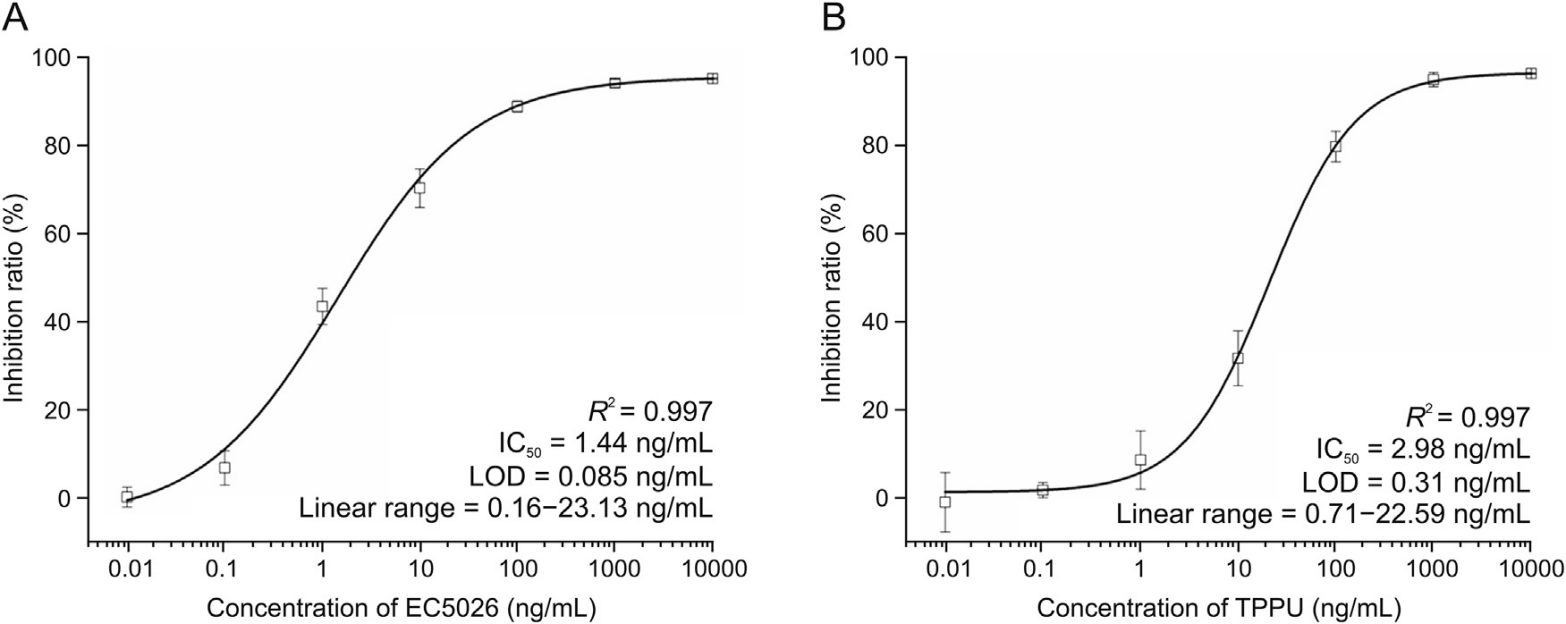
(A) The standard curve of enzyme-linked immunosorbent assay (ELISA) toward EC5026 standards with Nb-EC03. (B) The standard curve of ELISA toward TPPU standards with Nb-TP02. The error bar represents the standard deviation (*n* = 3).

Under best experimental factors, various concentrations of sEHI (EC5026 and TPPU) standard solutions were used to evaluate the sensitivity of the Nb-based immunoassay. For competitive immunoassays suitable for small molecule analytes, the output signal is inversely proportional to the concentration of the analyte. The resulting competitive inhibition curves for EC5026 and TPPU are shown in Figs. 5A and B, respectively. The four-parameter logistic equation for the EC5026 immunoassay was *y* = 95.28–100.09/[1 + (*x*/1.44)ˆ0.63] with *R*^2^ of 0.997, the IC_50_ value of 1.44 ng/mL, LOD of 0.085 ng/mL and a linear range of 0.16–23.13 ng/mL. The four-parameter logistic equation for the TPPU immunoassay was *y* = 97.11–97.43/[1 + (*x*/2.98)ˆ0.96] with *R*^2^ of 0.997, the IC_50_ value of 2.98 ng/mL, LOD of 0.31 ng/mL and the linear range of 0.71–22.59 ng/mL.

### 3.7 CR

A series of structurally similar chemicals were utilized for CR investigation to identify specific epitopes of antibody-producing haptens. Cross reacting compound **8** is under development as a veterinary pharmaceutical (Guedes #1077) [31]. Nb-EC03 and Nb-TP02 were raised against immunogens that exposed the furthest site of haptens when conjugated to Thy via the amine moiety (Fig. 1). Therefore, Nb-EC03 and Nb-TP02 will be highly selective to the furthest site (part a, −(OCF_3_)phenyl group), lower selectivity to the middle left site (part b, urea skeletal structure), and little or no selectivity to the middle right site (part c, piperidine) and the nearest site (part d, tail). This hypothesis is supported by the following analysis. For part a, as listed in Table 1, chemicals containing the −(OCF_3_)phenyl group (compounds **M1**, **M2**, and **7−12**) strongly inhibited the assay, resulting in highly sensitive detection and a CR in the range of 31.6%–104.3% compared to EC5026, and 25.6%−100% compared to TPPU. In contrast, analytes lacking −(OCF_3_)phenyl group (compounds **21** and **22**) produced CR barely above 0.2%, suggesting that Nb-EC03 and Nb-TP02 are selective to the −(OCF_3_)phenyl group. It should be noted that the adamantyl group in these compounds is a hydrophobic rigid cyclic ring. Although it can yield high potency by binding to the target sEH enzyme, its structure precludes sensitive detection with these nanobodies. Further, the presence or absence of F at the 3-position of the phenyl group had no significant effect on the CR, indicating that F is not a key factor for nanobody recognition although it improves inhibitor potency on the human sEH enzyme. This is expected based on the very small size of F. Obviously, low CR values (<1.0%) were produced when phenyl group has no substituent or a different substituent from −(OCF_3_) group at the *para*−position (compounds **16−20**), suggesting that Nb-EC03 and Nb-TP02 are highly selective to the presence of the −(OCF_3_) group. For part b of EC5026 and TPPU, the CR value of the compound with amide skeletal structure (compound **13**) was around 7.5%, which was much lower than that of urea as the skeleton structure, meaning that Nb-EC03 and Nb-TP02 show lower selectivity to the middle left site of molecule (part b). For part c of EC5026 and TPPU, compound **8** without a piperidine in part c could still produce a high CR value of 63.4% with Nb-EC03 and 75.0% with Nb-TP02, indicating that Nb-EC03 and Nb-TP02 show little selectivity to the middle right site of the molecule (part c). For part d of EC5026 and TPPU, high CR values (34.3%−104.3%) were produced when the structure of part d is different from that of EC5026 and TPPU (**M1**, **M2** and compounds **7−12**), indicating that Nb-EC03 and Nb-TP02 show no selectivity to part d. In addition, starting materials of synthesis (compounds **14** and **15**) show very low CR values, which indicated that Nb would fail to recognize −(OCF_3_) group if parts b, c and d were absent despite the low selectivity of Nbs to parts b, c and d. The corresponding anilines (compounds **14** and **15**) have never been detected as a metabolite of either EC5026 or TPPU [10,32], and they have no activity on the target sEH enzyme (IC_50_ > 50,000 nM), and the lack of significant binding is important to note. The structure activity relationships (SAR) seen with the biological target (sEH enzyme) is clearly different from the SAR with the nanobodies tested here. The nanobodies show high sensitivity for 1,3-disubstituted ureas very similar to EC5026 and TPPU. However, the SAR demonstrates that the apparent high affinity of the nanobodies for compounds closely related to EC5026 is not based on the same molecular characteristics that lead to high affinity binding of small molecules to the sEH active site [33]. Thus, using the resulting nanobody assays as a surrogate for binding to and inhibiting the sEH enzyme is not warranted. The SAR demonstrated in Table 1 showing a high level of selectivity for the structures of EC5026 and TPPU, allows users of the assay to predict possible cross reacting substances and particularly other pharmaceuticals that could cross react, while showing a very different SAR from that seen with the rodent or human sEH [33]. Such selectivity studies are valuable in identifying possible cross reacting substances.

**Table 1:** Cross-reactivity (CR) of nanobodies (Nb)-based enzyme-linked immunosorbent assays (ELISAs) for determination of EC5026 and TPPU.

Name	Chemical structure	EC5026 immunoassay IC_50_ (ng/mL)	CR (%)	TPPU immunoassay IC_50_ (ng/mL)	CR (%)
EC5026		1.44	100	3.44	86.6
TPPU		2.72	52.9	2.98	100
M1 (The metabolite of EC5026)		1.47	98.0	4.10	72.7
M2 (The metabolite of EC5026)		1.38	104.3	5.01	59.4
7		1.85	77.8	3.67	81.2
8		2.27	63.4	3.97	75.0
9		2.56	56.2	5.54	53.8
10		4.20	34.3	5.89	50.6
11		4.23	34.0	8.91	33.4
12		4.46	31.6	11.66	25.6
13		18.46	7.8	41.95	7.1
14		>1.5 × 10^5^	<0.01	>3.0 × 10^5^	<0.01
15		>1.5 × 10^5^	<0.01	>3.0 × 10^5^	<0.01
16		1.6 × 10^3^	0.09	1.2 × 10^4^	0.02
17		1.44 × 10^4^	0.01	>2.4 × 10^5^	<0.01
18		378.95	0.4	1.2 × 10^3^	0.2
19		>1.5 × 10^5^	<0.01	>2.4 × 10^5^	<0.01
20		>1.5 × 10^5^	<0.01	>2.4 × 10^5^	<0.01
21		>1.5 × 10^5^	<0.01	1.3 × 10^3^	0.2
22		>1.5 × 10^5^	<0.01	2.3 × 10^3^	0.1

Careful design and excellent organic synthesis of haptens enable the development of a diverse but closely related chemical libraries that can be exploited to generate nanobodies with required features: target-, group-, or class-specific reagents. Although EC5026 was metabolized in vivo after administration in mice, rats, dogs and humans, most of the predicted and identified metabolites were present at very low concentration, and showed little or no inhibition of the sEH [10]. However, as one would predict from structures of EC5026, metabolites M1 and M2 were produced in significant amounts and were potent inhibitors of the sEH. As predicted from the design of the immunizing hapten in our study, Nb-EC03 recognized not only EC5026 but also its metabolites (M1 and M2) with a CR value around 100%. Therefore, Nb-EC03 could be used for the construction of ELISA or biosensors that would be able to monitor the total level of EC5026 and its two principle biologically active metabolites at the same time instead of separately. This will improve the efficacy of the assay and reduce analytical costs.

### 3.8 Matrix tolerance and spike-recovery study

Sample preparation is an essential step for the analysis of complex samples, which affects the accuracy of many analytical methods. Dilution is the primary and simplest mode to reduce matrix interference in ELISA. In this work, the urine collected from a healthy volunteer and the commercially available FBS were used to study the matrix interference on Nb-based ELISA performance. As demonstrated in Figs. S7A and B, there was no significant change in the sensitivity (IC_50_ value) and *A*_max_ when the assay was performed in the range of 5%–20% human urine compared to the assay conducted in buffer (0%), suggesting that the matrix effect could be essentially eliminated by dilution. However, a constant decrease in *A*_max_ and an increase in IC_50_ value were observed when the content of urine was over 20%, suggesting that urine samples should be diluted a minimum of 5-fold to reduce matrix interference. Thus, the high sensitivity of the assay allows dilution to be used to eliminate matrix effects of human urine rather than the added expense of partitions or chromatographic clean up. Here, 10-fold dilution of human urine was selected as in most publications [34]. A similar analysis was performed with the serum matrix for EC5026 and TPPU assays. An increase in IC_50_ value and a constant decrease in *A*_max_ value were found when the content of serum was 2%–10% (Figs. S7C and D). Thus, a minimum of 10-fold dilution of serum samples with assay buffer was chosen for further investigations.

Precision and accuracy tests were performed to evaluate the feasibility of the established Nb-based ELISAs for EC5026 and TPPU determination, respectively. EC5026 and TPPU standard solutions were spiked into 10-fold diluted human urine and FBS with high, medium and low spiked levels (20, 5, and 1.25 ng/mL), respectively. As listed in Table 2, the precision (shown as CV%) for EC5026 spiked in urine and FBS sample was under 10.9% both intra-assay and inter-assay, and the accuracy of intra-assay and inter-assay (*n* = 7) ranged from 96.8%–111.4% and 100.2%–104.8%, respectively. The precision (shown as CV%) for TPPU spiked in urine and FBS sample was under 8.3% both intra-assay and inter-assay, and the accuracy of intra-assay and inter-assay (*n* = 7) ranged from 98.2% to 104.3% and 97.6%–104.0%, respectively. Overall, the accuracy was above 96.8% and the CV value was below 10.9%, demonstrating that the established Nb-based ELISAs could effectively and accurately detect the concentration of EC5026 and TPPU in urine and serum samples.

**Table 2:** Accuracy and precision analysis of EC5026 and TPPU in spiked samples by nanobodies (Nb)-based enzyme-linked immunosorbent assay (ELISA).

Analyte	Matrix	Spiked concentration (ng/mL)	Intra-assay			Inter-assay		
			Found concentration (mean ± SD, ng/mL)	Precision (CV%)	Accuracy (%)	Calculated concentration (mean ± SD, ng/mL)	Precision (CV%)	Accuracy (%)
EC5026	Urine	20	20.21 ± 2.20	10.9	100.1	20.38 ± 0.48	2.3	101.9
		5	4.84 ± 0.42	8.7	96.8	5.01 ± 0.16	3.2	100.2
		1.25	1.31 ± 0.11	8.4	104.8	1.26 ± 0.05	4.0	100.8
	FBS	20	22.29 ± 1.93	8.6	111.4	20.95 ± 1.10	5.3	104.8
		5	5.17 ± 0.37	7.2	103.4	5.16 ± 0.17	3.3	103.2
		1.25	1.26 ± 0.07	5.6	100.8	1.27 ± 0.03	2.4	101.6
TPPU	Urine	20	20.85 ± 1.73	8.3	104.3	20.27 ± 0.29	1.4	101.4
		5	4.91 ± 0.34	6.9	98.2	5.01 ± 0.24	4.8	100.2
		1.25	1.27 ± 0.10	7.9	101.6	1.24 ± 0.04	3.2	99.2
	FBS	20	20.58 ± 0.88	4.3	102.9	20.79 ± 1.03	5.0	104.0
		5	5.04 ± 0.22	4.4	100.8	4.96 ± 0.10	2.0	99.2
		1.25	1.26 ± 0.08	6.3	100.8	1.22 ± 0.09	7.4	97.6

FBS: fetal bovine serum; CV: coefficient variation.

### 3.9 Application in clinical samples

A comparative study was performed to compare Nb-based ELISA and LC-MS analysis using urine samples collected from a human clinical trial (NCT04228302) with EC5026. EC5026 and its biologically active metabolites (M1 and M2) in the urine samples were quantified by LC-MS, respectively. The Nb-based ELISA was used to test the level of equivalent amount of EC5026 in the selected samples. As seen in Table 3 [35], there is a good agreement between the level of equivalent EC5026 detected by the Nb-based ELISA and the total content of EC5026, M1, and M2 detected by LC-MS (*R*^2^ = 0.987 and accuracy = 98.9%), indicating that Nb-based ELISA could be used for simultaneously quantitative monitoring of sEHI and their bioactive metabolites in complex matrices. In addition, the Nb-based ELISA could be applied to evaluate the combined inhibitory potency of the parent EC5026 and bioactive metabolites M1 and M2. It is worth mentioning that in practical testing, due to the fact that most of EC5026 are metabolized into M1 and M2, simply detecting EC5026 may not provide a comprehensive guidance on pharmaceutical investigations (barely no correlation between EC5026 and inhibitory potency). When designing an immunoassay and monitoring the pharmaceutical level of drugs, it may be necessary to consider the detection of metabolites in the clinical samples. Consequently, immunoassay based on the Nb-EC03 and Nb-TP02 have the potential to be an excellent pharmaceutical analysis tools for evaluation of inhibitory potency and quantitative monitoring of structurally similar sEHI and their metabolites during clinical trials to quickly measure exposure and effective dose.

**Table 3:** Comparison of liquid chromatography mass spectrometry (LC-MS) and nanobodies (Nb)-based enzyme-linked immunosorbent assay (ELISA) to detect EC5026 and its major metabolites in relation to biological potency efficacy in human urine samples after exposure to EC5026.

Sample	Time post exposure (h)	LC-MS analysis				Nb-based ELISA analysis	Theoretical inhibitory potency (kP.U.)^a^
		[EC5026] (ng/mL)	[M1] (ng/mL)	[M2] (ng/mL)	Σ[EC5026, M1, M2] (ng/mL)	Equivalent [EC5026] (ng/mL) ^#^	
Subject 1	0–8	2.96	24.22	28.66	55.85	54.12	1.49
	8–16	1.41	44.30	61.59	107.31	108.17	2.70
	16–24	1.81	35.82	51.04	88.67	80.10	2.26
	24–32	1.43	48.89	67.21	117.53	114.74	2.95
	32–40	1.63	36.78	47.44	85.86	77.98	2.18
	40–48	1.24	33.49	43.94	78.67	71.98	1.99
Subject 2	0–8	3.01	12.48	12.99	28.48	32.03	0.81
	8–16	5.57	38.41	53.46	97.44	94.16	2.61
	16–24	4.86	43.37	59.34	107.57	113.83	2.84
	24–32	2.84	35.76	45.69	84.29	80.62	2.19
	32–40	3.46	55.70	75.89	135.05	132.00	3.46
	40–48	2.01	40.74	52.38	95.14	94.94	2.42
Subject 3	0–8	6.53	13.41	14.93	34.87	31.25	1.10
	8–16	4.69	36.41	50.31	91.41	102.52	2.43
	16–24	2.59	67.03	106.12	175.74	174.26	4.44
	24–32	2.98	31.25	44.20	78.43	88.42	2.05
	32–40	2.06	67.62	103.57	173.25	166.56	4.35
	40–48	1.18	98.28	158.70	258.15	243.31	6.42
Comparison LC-MS to ELISA	*r*^2^	0.123	0.980	0.984	0.987	–	–
	Overall accuracy (%)	4.1	41.5	56.0	98.9	–	–
Comparison to inhibitory potency	Correl.	−0.347	0.995	0.998	0.999	0.994	–
	Spearman ranking ρ	−0.243	0.973	0.990	0.994	0.977	–

^#^Nb-based ELISA detects equivalently EC5026, M1 and M2. Because the standard curve was based on EC5026 standard solution, results are reported as equivalents of EC5026.

a 1 Potency unit (P.U) is equal to the concentration of EC5026 permitting the inhibition of 50% of 1 nM of the human sEH in vitro using the assay described by Jones et al. [35]. 1PU = 0.0162 ng/mL of EC5026 = 0.0405 ng/mL of M1 = 0.0405 ng/mL of M2. The theoretical inhibitory potency was calculated based on the concentrations obtained by LC-MS.

## 4 Conclusion

In summary, high-affinity Nb-EC03 and Nb-TP02 against sEHI, EC5026 and TPPU were isolated from a phage display library derived from an immunized llama. Using optimal Nbs and cAgs, indirect competitive ELISAs for the determination of EC5026, TPPU and the bioactive metabolites of EC026 were established. The Nb-based ELISAs for EC5026 and TPPU showed excellent precision (shown as CV%, <10.9%) and accuracy (>96.8%) in spiked human urine and FBS samples. Furthermore, the proposed Nb-based ELISA could be utilized to quantify the total level of EC5026 and its major biologically active metabolites in clinical dosed human urine samples, and could evaluate inhibitory potency. Thus, the assay can serve as an alternative or complementary method for experimental research or clinical testing such as in the prediction of target engagement and in vivo efficacy. Besides sEHI, the type of assay described herein could be used for the analysis of other small molecule targets. Immunochemistry probably is the most versatile of modern analytical techniques but the application of this technology should be based on multiple parameters including the clinical and regulatory needs of the analytical chemist, resources available to the laboratory and of course the target analyte.

The importance of immunoassays to the practice of clinical chemistry, even for small molecules, was illustrated by the award of the 1977 Nobel Prize to Dr. Rosalyn Yalow. However, due in part to the ephemeral nature of antibodies, LC-MS has largely replaced immunoassays for clinical analysis of small molecules. Nowadays, the availability of stable and “immortal” nanobody may lead to a resurgence in the use of immunoassays for small molecules in clinical chemistry as it is illustrated here with the detection of sEHI. Such technology should be complementary to LC-MS for the rapid detection of pharmaceuticals by the patient bedside, in field samples in veterinary medicine or with pharmaceutical investigations in developing countries which is not always possible with LC-MS. Finally, the low cost of nanobody production and stability should be attractive for affinity chromatography, sample concentration and possibly even for therapeutic uses such as dialysis, as antidotes for over dose, modifiers for drug delivery as in parallel nanobodies are developed directly for a variety of therapies themselves.

## CRediT author statement

**Huiyi Yang**: Investigation, Methodology, Data curation, Writing - Original draft preparation, Funding acquisition; **Meng Qi**: Investigation and Methodology; **Qiyi He**: Investigation and Methodology; **Sung Hee Hwang**: Investigation, Methodology, Writing - Reviewing and Editing; **Jun Yang**; Methodology, Writing - Reviewing and Editing; **Mark McCoy**: Methodology; **Christophe Morisseau**: Conceptualization, Writing - Reviewing and Editing; **Suqing Zhao**: Funding acquisition; **Bruce D. Hammock**: Writing - Reviewing and Editing, Funding acquisition and Supervision.

## Declaration of competing interest

S.H.H. and J.Y. are part-time employees and B.D.H. is the cofounder of EicOsis LLC, a company that is developing sEH inhibitors for the treatment of neuropathic pain. S.H.H., J.Y., C.M. and B.D.H. are inventors on patents held by the University of California related to pharmaceutical usage of sEH inhibitors. The other authors declare that they have no conflict of interests.
