# Supplementary material for: Quantification of soluble epoxide hydrolase inhibitors in experimental and clinical samples using the nanobody-based ELISA
Source: J Pharm Anal. 2023 May 16;13(9):1013–23. doi: 10.1016/j.jpha.2023.05.006 (PMC10568103; doi:10.1016/j.jpha.2023.05.006)
Supplement: Multimedia component 1 [file mmc1.docx]

**Supporting Information**

**Quantification of soluble epoxide hydrolase inhibitors in experimental and clinical samples using the nanobody-based ELISA**

Huiyi Yang^a,b^; Meng Qi^b,c^; Qiyi He^b^; Sung Hee Hwang^b^; Jun Yang^b^; Mark McCoy^b^; Christophe Morisseau^b^; Suqing Zhao^a,^*; Bruce D. Hammock^b,^**

*^a^Department of Pharmaceutical Engineering, School of Biomedical and Pharmaceutical Sciences, Guangdong University of Technology, Guangzhou, People’s Republic of China*

*^b^Department of Entomology and Nematology and UCD Comprehensive Cancer Center, University of California, Davis, Davis, California 95616, United States*

*^c^Langfang Normal University, Langfang, People’s Republic of China*

*** First Corresponding Author:**

Professor Suqing Zhao

E-mail address: [sqzhao@gdut.edu.cn](mailto:sqzhao@gdut.edu.cn) (S. Zhao)

**** Second Corresponding Author:**

Professor Bruce D. Hammock

E-mail address: bdhammock@ucdavis.edu (B.D. Hammock)

E-mail addresses of other authors:

iyiuhgnay@163.com (H. Yang); menqi@ucdavis.edu (Q. Meng);

qiyhe@ucdavis.edu (Q. He); shhwang@ucdavis.edu (S.H. Hwang);

junyang@ucdavis.edu (J. Yang); mrmccoy@ucdavis.edu (M. McCoy);

chmorisseau@ucdavis.edu (C. Morisseau).

**Table of contents**

**Methods**

1. Synthesis of haptens

2. Synthesis of metabolites

3. Preparation of immunogens and coating antigens

4. Construction of the phage display library and screening of nanobodies

5 Expression, purification, and characterization of sEHI Nbs

6. Clinical urine samples analysis by UPLC-MS/MS

**Figures and tables**

**Fig. S1** Schematic alignment of deduced amino acid sequences of plasmids for nanobody against (A) EC5026 and (B) TPPU. (C) The binding affinity of clones EC02, EC03, EC04, EC06 and EC09 to different concentrations of EC5026. (D) The binding affinity of clones TP01, TP02, TP03, TP04 to different concentrations of TPPU.

**Fig. S2** Characterization of nanobodies against sEH inhibitors by SDS-PAGE

**Fig. S3** Optimization of concentrations of coating antigens and Nb-EC03 for the Nb-based ELISA with different coating antigens

**Fig. S4** Optimization of concentrations of coating antigens and Nb-TP02 for the Nb-based ELISA with different coating antigens

**Fig. S5** Standard curves of EC5026 with different physicochemical parameters and the relationship between three indicators *A_max_*, IC_50_ and *A_max_*/IC_50_ and physicochemical parameters of Nb-based ELISA for EC5026

**Fig. S6** Standard curves of TPPU with different physicochemical parameters and the relationship between three indicators *A_max_*, IC_50_ and *A_max_*/IC_50_ and physicochemical parameters of Nb-based ELISA for TPPU

**Fig. S7** Calibration curves for EC5026 and TPPU in varying concentrations of human urine and fetal bovine serum

**Table S1** Panning conditions

**Table S2** Optimization results of the concentration of coating antigens and nanobodies

**Table S3** Nb-EC03 based ELISA results for homologous and heterologous coating antigens

**Table S4** Nb-TP02 based ELISA results for homologous and heterologous coating antigens

**Methods**

**1. Synthesis of haptens**

**General**. All reagents and solvents were obtained from commercial suppliers and were used without further purification. All reactions, unless otherwise described, were performed under an inert atmosphere of dry nitrogen. Melting points were determined on an OptiMelt melting point apparatus and were uncorrected. ^1^H NMR spectra were recorded at 400 or 600 MHz, respectively. Optical rotations were determined at Robertson Microlit laboratories, Ledgewood, NJ. Mass spectra were measured by LC-MS equipped with an Agilent 1260 and a Bruker timsTOF Pro using electrospray (-) ionization. Source parameters were -4.20 kV capillary voltage, nebulizer gas at 2 bar, dry gas flow at 10 L/min, and temperature at 200 ℃. The mass spectrometer was calibrated against sodium formate before acquiring the data to provide an accurate mass. The mobile phases are water with 0.1% acetic acid (A) and acetonitrile with 0.1% acetic acid (B). The gradient was from 30% B and increased to 90% B in 3 min. The separation was run on a Phenomenex Kinetex, 30 x 2 mm 1.7 um C18 column. Flash chromatography was performed on silica gel. The starting materials 1-(3-fluoro-4-(trifluoromethoxy)phenyl)-3-(piperidin-4-yl)urea (compound **11**) and1-(piperidin-4-yl)-3-(4-trifluoromethoxy)phenyl) urea (compound **12**) were prepared by literature methods [10,11].

**General procedure for the synthesis of compound** **1-1, 2-1, 4-1 and 5-1.**

To a solution of an amine (1 mmol) and an acid (1.2 eq) in DMF (10 mL) were added PyBOP (1.3 eq) followed by DIPEA (1.5 eq) at 0 ^o^C. The reaction mixture was warmed to room temperature. After stirring overnight, 40 mL of ethyl acetate was added. The organic solvent was washed with water three times and dried with MgSO_4_. After removing the solvent in vacuo, the crude product was purified by column chromatography using 60% EtOAc/hexanes as an eluent to obtain the desired product.

**The preparation of (±)-1-(3-fluoro-4-(trifluoromethoxy)phenyl)-3-(1-(2-methyl-3-(4-nitrophenyl)propanoyl)piperidin-4-yl)urea (Compound 1-1).**

The general method above was used with 1-(3-fluoro-4-(trifluoromethoxy)phenyl)-3-(piperidin-4-yl)urea and (±)-2-methyl-3-(4-nitrophenyl)propanoic acid to afford a pale-yellow solid (0.30 g, 61% yield). mp 78.0 -80.9 ^o^C. ^1^H NMR (400 MHz, DMSO-*d*_6_) δ 8.73 (d, *J* = 23 Hz, 1H), 8.14 (dd, *J* = 8 and 6 Hz, 2H), 7.65 (dd, *J* = 13 and 3 Hz, 1H), 7.49 (t, *J* = 8 Hz, 2H), 7.38 (t, *J* = 9 Hz, 1H), 7.10 (d, *J* = 9 Hz, 1H), 6.31 (dd, *J* = 34 and 8 Hz, 1H), 4.16 (t, *J* = 14 Hz, 1H), 3.83 (d, *J* = 14 Hz, 1H), 3.72-3.59 (m, 1H), 3.27-2.94 (m, 3H), 2.81-2.66 (m, 2H), 1.82-1.69 (m, 2H), 1.35-1.14 (2H), 1.02 (dd, *J* = 7 and 3 Hz, 3H). HRMS (*m/z*): [M-1]^-^ calcd for C_23_H_24_F_4_N_4_O_5_, 511.1605; found, 511.1608.

**The preparation of (±)-1-(3-fluoro-4-(trifluoromethoxy)phenyl)-3-(1-(2-methyl-5-(4-nitrophenyl)pentanoyl)piperidin-4-yl)urea (Compound 2-1).**

The general method above was used with 1-(3-fluoro-4-(trifluoromethoxy)phenyl)-3-(piperidin-4-yl)urea and (±)-2-methyl-5-(4-nitrophenyl)pentanoic acid to afford a pale-yellow solid (0.32 g, 60% yield). mp 146.2 -148.8 ^o^C. ^1^H NMR (400 MHz, DMSO-*d*_6_) δ 8.75 (d, *J* = 15 Hz, 1H), 8.15 (d, *J* = 7 Hz, 2H), 7.66 (dd, *J* = 13 and 3 Hz, 1H), 7.48 (dd, *J* = 9 and 3 Hz, 2H), 7.39 (t, *J* = 9 Hz, 1H), 7.15 – 7.07 (m, 1H), 6.35 (d, *J* = 8 Hz, 1H), 4.26-4.16 (m, 1H), 3.86 (d, *J* = 13.6 Hz, 1H), 3.76-3.64 (m, 1H), 3.14 (t, *J* = 13 Hz, 1H), 2.86-2.64 (m, 4H), 1.89-1.73 (m, 2H), 1.63-1.49 (m, 3H), 1.35-1.13 (m, 3H), 0.97 (t, *J* = 7 Hz, 3H). HRMS (*m/z*): [M-1]^-^ calcd for C_25_H_28_F_4_N_4_O_5_, 539.1918; found, 539.1917.

**The preparation of (±)-1-(1-(2-methyl-5-(4-nitrophenyl)pentanoyl)piperidin-4-yl)-3-(4-(trifluoromethoxy)phenyl)urea (Compound 4-1).**

The general method above was used with 1-(piperidin-4-yl)-3-(4-trifluoromethoxy)phenyl) urea and (±)-2-methyl-5-(4-nitrophenyl)pentanoic acid to afford a pale-yellow solid (0.37 g, 70% yield). mp 146.7 -149.5 ^o^C. ^1^H NMR (400 MHz, DMSO-*d*_6_) δ 8.52 (d, *J* = 14 Hz, 1H), 8.25 – 8.09 (m, 2H), 7.51-7.44 (m, 4H), 7.21 (d, *J* = 9 Hz, 2H), 6.24 (d, *J* = 8 Hz, 1H), 4.25-4.15 (m, 1H), 3.86 (d, *J* = 14 Hz, 1H), 3.75-3.65 (m, 1H), 3.15 (t, *J* = 13 Hz, 1H), 2.88-2.66 (m, 4H), 1.92-1.75 (m, 2H), 1.63-1.50 (m, 3H), 1.35-1.12 (m, 3H), 0.97 (t, *J* = 7 Hz, 3H). HRMS (*m/z*): [M-1]^-^ calcd for C_25_H_29_F_3_N_4_O_5_, 521.2011; found, 521.2013.

**The preparation of (±)-1-(1-(2-methyl-3-(4-nitrophenyl)propanoyl)piperidin-4-yl)-3-(4-(trifluoromethoxy)phenyl)urea (Compound 5-1).**

The general method above was used with 1-(3-fluoro-4-(trifluoromethoxy)phenyl)-3-(piperidin-4-yl)urea and (±)-2-methyl-3-(4-nitrophenyl)propanoic acid to afford a pale-yellow solid (0.30 g, 61% yield). mp 74.5 -78.3 ^o^C. ^1^H NMR (600 MHz, DMSO-*d*_6_) δ 11.98 (^1^H NMR (400 MHz, DMSO-*d*_6_) δ 8.51 (d, *J* = 22 Hz, 1H), 8.14 (dd, *J* = 9 and 7 Hz, 2H), 7.53 – 7.40 (m, 4H), 7.21 (d, *J* = 9 Hz, 2H), 6.19 (dd, *J* = 36 and 8 Hz, 1H), 4.15 (t, *J* = 14 Hz, 1H), 3.82 (d, *J* = 14 Hz, 1H), 3.72-3.58 (m, 1H), 3.28-2.94 (m, 3H), 2.80-2.65 (m, 2H), 1.82-1.69 (m, 2H), 1.34-1.14 (m, 2H), 1.03 (dd, *J* = 7 and 3 Hz, 3H). HRMS (*m/z*): [M-1]^-^ calcd for C_23_H_25_F_3_N_4_O_5_, 493.1699; found, 493.1709.

**General procedure for the synthesis of compounds** **1, 2, 4 and 5**. To a solution of nitro compounds 1–1, 2–1, 4–1 or 5–1 (0.94 mmol) in EtOH (10 mL) was added tin (II) chloride dihydrate (5 eq) at room temperature. The reaction mixture was warmed to 70^o^C and stirred overnight. After the reaction was completed, the reaction mixture was cooled to room temperature. Twenty milliliters of saturated aqueous NaHCO_3_ solution was added to basify the reaction mixture. After evaporating EtOH in vacuo, the remained mixture was extracted with EtOAc three times, and the combined organic layers were dried with NaSO_4_. After removing the solvent in vacuo, the crude product was purified by column chromatography with 70% EtOAc/hexanes as eluent to obtain the desired product.

**The preparation of (±)-1-(1-(3-(4-aminophenyl)-2-methylpropanoyl)piperidin-4-yl)-3-(3-fluoro-4-(trifluoromethoxy)phenyl)urea (Compound 1).**

The general method above was used with (±)-1-(3-fluoro-4-(trifluoromethoxy)phenyl)-3-(1-(2-methyl-3-(4-nitrophenyl)propanoyl)piperidin-4-yl)urea (Compound 1-1) to afford a white solid (0.27 g, 60% yield). mp 56.0 -60.2 ^o^C. ^1^H NMR (600 MHz, DMSO-*d*_6_) δ 8.77 (s, 1H), 7.66 (d, *J* = 13 Hz, 1H), 7.39 (t, *J* = 9 Hz, 1H), 7.10 (d, *J* = 9 Hz, 1H), 6.82 (dd, *J* = 16 and 8 Hz, 2H), 6.50 – 6.41 (m, 2H), 6.26 (dd, *J* = 122 and 8 Hz, 1H), 4.84 (s, 2H), 4.16 (dd, *J* = 49 and 13 Hz, 1H), 3.82-3.71 (m, 1H), 3.69-3.58 (m, 1H), 3.25-2.91 (m, 2H), 2.80-2.58 (m, 2H), 2.39-2.28 (m, 1H), 1.79-1.60 (m, 2H), 1.32-1.16 (m, 2H), 0.95 (dd, *J* = 10 and 6 Hz, 3H). HRMS (*m/z*): [M-1]^-^ calcd for C_23_H_26_F_4_N_4_O_3_, 481.1863; found, 481.1878.

**The preparation of (±)-1-(1-(5-(4-aminophenyl)-2-methylpentanoyl)piperidin-4-yl)-3-(3-fluoro-4-(trifluoromethoxy)phenyl)urea (Compound 2).**

The general method above was used with (±)-1-(3-fluoro-4-(trifluoromethoxy)phenyl)-3-(1-(2-methyl-5-(4-nitrophenyl)pentanoyl)piperidin-4-yl)urea (Compound 2-1) (0.51 g, 0.94 mmol) to afford a white solid (0.26 g, 55% yield). mp 70.2 -74.6 ^o^C. ^1^H NMR (400 MHz, DMSO-*d*_6_) δ 8.75 (d, *J* = 13 Hz, 1H), 7.67 (dd, *J* = 14 and 3 Hz, 1H), 7.39 (t, *J* = 9 Hz, 1H), 7.14 – 7.07 (m, 1H), 6.81 (dd, *J* = 8 and 5 Hz, 2H), 6.52 – 6.43 (m, 2H), 6.35 (d, *J* = 8 Hz, 1H), 4.79 (s, 2H), 4.26-4.14 (m, 1H), 3.84 (d, *J* = 14 Hz, 1H), 3.76-3.65 (m, 1H), 3.18-3.06 (m, 1H), 2.82-2.70 (m, 2H), 2.43-2.29 (m, 2H), 1.89-1.72 (m, 2H), 1.62-1.36 (m, 2H), 1.34-1.13 (m, 4H), 0.95 (dd, *J* = 9 and 6 Hz, 3H).HRMS (*m/z*): [M-1]^-^ calcd for C_25_H_30_F_4_N_4_O_3_, 509.2176; found, 509.2189.

**The preparation of (±)-1-(1-(5-(4-aminophenyl)-2-methylpentanoyl)piperidin-4-yl)-3-(4-(trifluoromethoxy)phenyl)urea (Compound 4).**

The general method above was used with (±)-1-(1-(2-methyl-5-(4-nitrophenyl)pentanoyl)piperidin-4-yl)-3-(4-(trifluoromethoxy)phenyl)urea (Compound 4-1) to afford a white solid (0.28 g, 60% yield). mp 74.6 -78.1 ^o^C. ^1^H NMR (400 MHz, DMSO-*d*_6_) δ 8.52 (d, *J* = 13 Hz, 1H), 7.46 (d, *J* = 9 Hz, 2H), 7.21 (d, *J* = 9 Hz, 1H), 6.84 – 6.77 (m, 2H), 6.50 – 6.43 (m, 2H), 6.23 (d, *J* = 8 Hz, 1H), 4.79 (s, 2H), 4.25-4.12 (m, 1H), 3.83 (d, *J* = 14 Hz, 1H), 3.75-3.64 (m, 1H), 3.12 (t, *J* = 12.3 Hz, 1H), 2.84-2.70 (m, 2H), 2.43-2.29 (m, 2H), 1.88-1.73 (m, 2H), 1.61-1.08 (m, 7H), 0.99-0.91 (m, 3H). HRMS (*m/z*): [M-1]^-^ calcd for C_25_H_31_F_3_N_4_O_3_, 491.2270; found, 491.2293.

**The preparation of (±)-1-(1-(3-(4-aminophenyl)-2-methylpropanoyl)piperidin-4-yl)-3-(4-(trifluoromethoxy)phenyl)urea (Compound 5)**

The general method above was used with (±)-1-(1-(2-methyl-3-(4-nitrophenyl)propanoyl)piperidin-4-yl)-3-(4-(trifluoromethoxy)phenyl)urea (Compound 5-1) to afford a white solid (0.26 g, 59% yield). mp 70.2 -74.6 ^o^C. ^1^H NMR (599 MHz, DMSO-*d*_6_) δ 8.52 (d, *J* = 6 Hz, 1H), 7.44 (d, *J* = 9 Hz, 2H), 7.19 (d, *J* = 9 Hz, 2H), 6.79 (dd, *J* = 16 and 8 Hz, 2H), 6.44 (dd, *J* = 12 and 8 Hz, 2H), 6.11 (dd, *J* = 125 and 8 Hz, 1H), 4.81 (s, 2H), 4.13 (dd, *J* = 52 and 14 Hz, 1H), 3.79-3.52 (m, 2H), 3.22-2.87 (m, 2H), 2.79-2.55 (m, 2H), 2.35-2.24 (m, 1H), 1.77-1.58 (m, 2H), 1.30-1.12 (m, 2H), 0.92 (dd, *J* = 9.4, 6.4 Hz, 3H). HRMS (*m/z*): [M-1]^-^ calcd for C_23_H_27_F_3_N_4_O_3_, 463.1957; found, 463.2003.

**The preparation of 4-(4-(3-(3-fluoro-4-(trifluoromethoxy)phenyl)ureido)piperidin-1-yl)-4-oxobutanoic acid (Compound 3).**

To a solution of 1-(3-fluoro-4-(trifluoromethoxy)phenyl)-3-(piperidin-4-yl)urea (0.3 g, o.93 mmol) and succinic anhydride (0.09 mL, 1.1 eq) in THF (20 mL) were added DIPEA (0.24 mL, 1.5 eq) followed by a catalytic amount of DMAP at room temperature. After stirring the reaction mixture overnight, 20 mL of water was added. The organic solvent was removed in vacuo. The remaining precipitates were filtered and washed thoroughly with water. The filtered solid was recrystallized with EtOAc/hexanes to obtain the desired product (0.37 g, 94% yield). mp 166.5-167.9 ^o^C. ^1^H NMR (600 MHz, DMSO-*d*_6_) δ 11.98 (s, 1H), 8.75 (s, 1H), 7.63 (dd, *J* = 13 and3 Hz, 1H), 7.36 (t, *J* = 9 Hz, 1H), 7.07 (dt, *J* = 9 and 1 Hz, 1H), 6.34 (d, *J* = 8 Hz, 1H), 4.13 (d, *J* = 13 Hz, 1H), 3.76 (d, *J* = 14 Hz, 1H), 3.70-3.62 (m, 1H), 3.10 (t, *J* = 12 Hz, 1H), 2.75 (t, *J* = 13 Hz, 1H). 2.53-2.46 (m, 2H), 2.41-2.35 (m, 2H), 1.84-1.72 (m. 2H), 1.34-1.13 (m, 2H). ^.^HRMS (*m/z*): [M-1]^-^ calcd for C_17_H_19_F_4_N_3_O_5_, 420.1183; found, 420.1191.

**The preparation of 4-oxo-4-(4-(3-(4-(trifluoromethoxy)phenyl)ureido)piperidin-1-yl)butanoic acid (Compound 6).**

To a solution of 1-(piperidin-4-yl)-3-(4-trifluoromethoxy)phenyl) urea (0.3 g, 1 mmol) and succinic anhydride (0.09 mL, 1.1 eq) in THF (20 mL) were added DIPEA (0.26 mL, 1.5 eq) followed by a catalytic amount of DMAP at room temperature. After stirring the reaction mixture overnight, 20 mL of water was added. The organic solvent was removed in vacuo. The remaining precipitates were filtered and washed thoroughly with water. The filtered solid was recrystallized with EtOAc/hexanes to obtain the desired product (0.36 g, 90% yield). mp 179.4 -180.9 ^o^C. ^1^H NMR (600 MHz, DMSO-*d*_6_) δ 11.98 (s, 1H), 8.52 (s, 1H), 7.43 (d, *J* = 9 Hz, 2H), 7.18 (d, *J* = 9 Hz, 2H), 6.22 (d, *J* = 7 Hz, 1H), 4.12 (d, *J* = 13 Hz, 1H), 3.75 (d, *J* = 14 Hz, 1H), 3.70-3.62 (m, 1H), 3.10 (t, *J* = 12 Hz, 1H), 2.76 (t, *J* = 13 Hz, 1H), 2.54-2.46 (m, 2H), 2.42-2.35 (m, 2H), 1.85-1.72 (m, 2H), 1,34-1.12 (m, 2H). ^.^HRMS (*m/z*): [M-1]^-^ calcd for C_17_H_20_F_3_N_3_O_5_, 402.1277; found, 402.1292.

**2. Synthesis of metabolites**

**The preparation of 1-(3-fluoro-4-(trifluoromethoxy)phenyl)-3-(1-((2S,3R)-3-hydroxy-2-methylbutanoyl)piperidin-4-yl)urea (Compound M1).**

To a solution of 1-(3-fluoro-4-(trifluoromethoxy)phenyl)-3-(piperidin-4-yl)urea (1.0 g, 3.11 mmol) and (2*S*, 3*R*)-3-hydroxy-2-methylbutanoic acid (0.44 g, 3.74 mmol) in DMF (15 mL) were added PyBOP (1.94 g, 3.74 mmol) followed by Et_3_N (0.65 mL, 4.67 mmol) at 0 ^o^C. The reaction mixture was warmed to room temperature. After stirring overnight, 50 mL of EtOAc was added and the organic layer was washed twice with water. After drying the organic layer with MgSO_4_, the solvent was removed using a rotary evaporator. The crude product was purified by column chromatography (10% MeOH in EtOAc as eluent) to obtain the desired product (1.1 g, 83.9% yield) as a white solid. mp 45.3-47.2 ^o^C. ^1^H NMR (600 MHz, DMSO-*d*_6_) δ 8.75 (d, *J* = 33 Hz, 1H), 7.65 (d, *J* = 13 Hz, 1H), 7.37 (t, *J* = 9 Hz, 1H), 7.09 (d, *J* = 9 Hz, 1H), 6.37 (t, *J* = 9 Hz, 1H), 4.56 (dd, *J* = 22 and 5 Hz, 1H), 4.21 (dd, *J* = 25 and 13 Hz, 1H), 3.91 (br s, 1H), 3.74-3.61 (m, 2H), 3.19-3.07 (m, 1H), 2.83-2.69 (m, 2H),, 1.89-1.71 (m, 2H), 1.38-1.15 (m, 2H), 1.02 (dd, *J* = 15 and 6 Hz, 3H), 0.90 (dd, *J* = 12 and 7 Hz, 3H). HRMS (*m/z*): [M-1]^-^ calcd for C_18_H_23_F_4_N_3_O_4_, 420.1546; found, 420.1550. ${[\alpha]}_{D}^{25}$ = +10.56 (0.506 g/100 mL (EtOH))

**The preparation of 1-(3-fluoro-4-(trifluoromethoxy)phenyl)-3-(1-((2S,3S)-3-hydroxy-2-methylbutanoyl)piperidin-4-yl)urea (Compound M2).**

To a solution of 1-(3-fluoro-4-(trifluoromethoxy)phenyl)-3-(piperidin-4-yl)urea and (2*S*, 3*S*)-3-hydroxy-2-methylbutanoic acid (0.09 g, 0.74 mmol) in DMF (6 mL) were added PyBOP (0.42 g, 0.81 mmol) followed by Et_3_N (0.13 mL, 0.93 mmol) at 0 ^o^C. The reaction mixture was warmed to room temperature. After stirring overnight, 20 mL of EtOAc was added and the organic layer was washed twice with water. After drying the organic layer with MgSO_4_, the solvent was removed using a rotary evaporator. The residue was purified by column chromatography with 10% MeOH in EtOAc to obtain the desired product (0.21 g, 82.0% yield) as a white solid. mp 148.7 -153.1 ^o^C. ^1^H NMR (400 MHz, DMSO-*d*_6_) δ 8.75 (d, *J* = 20 Hz, 1H), 7.67 (dd, *J* = 13 and 3 Hz, 1H), 7.38 (t, *J* = 9 Hz, 1H), 7.11 (d, *J* = 9 Hz, 1H), 6.37 (s, 1H), 4.55 (br s, 1H), 4.22 (t, *J* = 15 Hz, 1H), 3.98 – 3.86 (m, 1H), 3.77-3.63 (m, 2H), 3.16 (t, *J* = 13 Hz, 1H), 2.84-2.71 (m, 2H), 1.93-1.74 (m, 2H), 1.42-1.14 (m, 2H), 1.04 (dd, *J* = 9 and 6 Hz, 3H), 0.92 (t, *J* = 7 Hz, 3H). HRMS (*m/z*): [M-1]^-^ calcd for C_18_H_23_F_4_N_3_O_4_, 420.1183; found, 420.1191. ${[\alpha]}_{D}^{25}$ = +11.60 (0.506 g/100 mL (EtOH))

**3. Preparation of immunogens and coating antigens**

*Diazotization method*. Haptens 1, 2, 4, and 5 were coupled to carrier protein with the available amine group of corresponding haptens. Briefly, each hapten (0.01 mmol) was dissolved in 15 μL of ethanol and then mixed with 0.5 mL of HCl (1.0 M) under an ice bath. Successively, 0.25 mL of sodium nitrite solution (0.2 M in water) and 0.2 mL of DMF were added drop by drop. After stirring for 10 min, the above solution was introduced to the carrier protein solution (10.0 mg of Thy, BSA, or OVA in 3 mL of pre–cold borate buffer (0.2 M, pH 9.0, containing 0.5 mL of DMF) and stirred for 30 min. Finally, the pH of the yellow solution was adjusted to pH 7.0, and the final solution was dialyzed and stored at –20 °C until being used.

*Active ester method.* Hapten 3 and 6 were conjugated to carrier proteins with the available carboxyl group of the haptens. Briefly, each hapten (0.01 mmol) was dissolved in 100 μL of DMF. DCC (0.015 mmol) and NHS (0.015 mmol) dissolved in DMF were added dropwise in the above solution, respectively. The formation of the mixed anhydride solution was allowed to proceed overnight at room temperature. The supernatant was then added dropwise to an ice–cold carrier protein solution (8.0 mg of BSA, or OVA in 1.0 mL of PBS) by stirring for 12 h at 4℃. This two-step procedure reduced the chance of direct reaction with the protein lysines to give a cyclohexyl urea conjugate. Finally, the mixture was dialyzed against PBS for 3 days at 4℃.

**4. Construction of the phage display library and screening of the nanobodies**

The development of a highly specific and extensive nanobody phage display library was described previously by our group. Typically, a three-year-old llama was immunized subcutaneously with the mixture of immunogens (1-Thy, 2-Thy, 4-Thy, and 5-Thy) and Freund’s adjuvant biweekly. Seven days after the fifth immunization, 100 mL of fresh blood was collected and used for the isolation of Peripheral Blood Mononuclear Cell (PBMC), which was utilized to extract total RNA and synthesize cDNA. The purified VHH digested with restriction enzyme *SfiI* was ligated into the pComb3XSS vector digested by *Sfi I* and then the ligation product was electrotransferred into competent ER2738 cells from *Escherichia coli.* All cells on Luria-Bertani and ampicillin agar plates (LB-AMP, 5 g/L yeast extract, 10 g/L tryptone, 10 g/L NaCl, supplemented with 100 μg/L ampicillin and 15 g/L agar) were scraped off and collected, and then were infected with the M13KO7 helper phage to construct an immunized VHH phage display library for panning.

The procedure of panning and screening of high-affinity EC5026 and TPPU nanobody clones was listed as follows. The conditions for each round of screening were listed in **Table S1**. Firstly, cAgs (1-BSA, 2-BSA, and 3-BSA for EC5026 nanobody clones; 4-BSA, 5-BSA, and 6-BSA for TPPU nanobody clones) with decreasing concentrations (10, 1, and 0.1 μg/mL) were coated in two wells at 4℃ overnight, respectively. After coating, wells were blocked with 250 μL of 5% skim milk in PBS for 1 h. After washing with 0.05% PBST (the mixture of PBS and 0.05% Tween 20), the VHH phage display library was mixed with 0.05% PBST containing 1% BSA, and then was introduced into two wells coated with each cAgs. After incubating for 2 h at 37 °C, plates were washed at increasing frequency (10, 15, and 20 times) with a series of increasing concentrations of PBST (0.05%, 0.1% and 0.5%) to remove the unbound phages, after which the bound phages were eluted and collected by incubation with decreasing concentration of EC5026 or TPPU (100, 10 and 1 μg/mL) in competitive elution mode in each round, respectively. Finally, the titer was tested with 10 µL of eluted phages in each round and then the remaining eluted phages were amplified and purified for the next round of panning.

**5** **Expression, purification, and characterization of sEHI Nbs**

The pComb3XSS–Nb–EC5026 and pComb3XSS–Nb–TPPU phagemids were extracted and separately transformed into TOP 10F’ competent cells by heat shock (42°C, 90 s). One of the individual clones was selected and cultivated in 10.0 mL of Super Broth medium containing of 50 μg∙mL^–1^ carbenicillin (SB-CA). 1.0 mL of the overnight culture was transferred into 100 mL of SB–CA medium and shaken at 37°C until the OD_600_ value reached about 0.8, followed by the addition of IPTG at a final concentration of 100 mM to express nanobodies. The bacterial pellets of the overnight culture were collected by centrifugation (8,000 *g*, 15 min) and were lysed with B-PER lysis buffer for 1h. The lysate supernatants were collected by centrifugation (13,000 *g*, 10 min), followed by purification by a Ni-NTA resin column with elution by 250 mM imidazole. Finally, the eluent (purified Nb) was dialyzed in PBS to remove imidazole, the nanobody analyzed by SDS-PAGE, and stored at –20℃ until use. The procedure of expression and purification for nanobodies against EC5026 and TPPU were the same, but the respective plasmids were used separately.

**6. Clinical urine samples analysis by LC-MS/MS**

The urine samples were analyzed by an LC-MS/MS method using Waters (Milford, MA, USA) Acquity UPLC system with Waters Xevo TQS tandem mass spectrometer. The separation was implemented on a Phenomenex Kinetex 150 x 2 mm C18 column. The mobile phases are 0.1 % of acetic acid in water as phase A, 0.1% of acetic acid in methanol as phase B. The gradient of the separation started from 50% of B and increased to 63% of B at 3.6 min then ramped to 95% at 4 min. The flow rate was 0.4 mL/min. The mass spectrometer was operated with negative electrospray ionization. The MRM transitions are 404.2/194 for EC5026, 420.2/194 for both M1 and M2. The source parameters of mass spectrometer are: capillary voltage: –3 KV, desolvation temperature: 300°C, desolvation gas flow: 800 L/hr; cone: 150 L/min; nebulizer: 7 bar.

**Figures and tables**

**
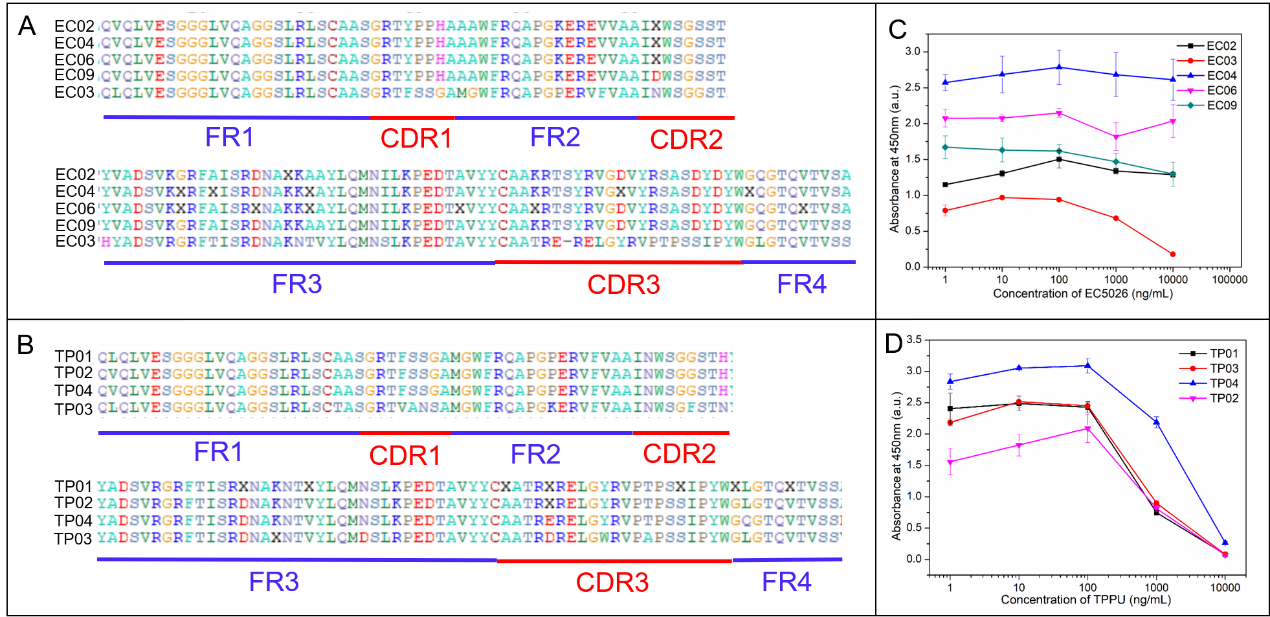
**

**Fig. S1** Schematic alignment of deduced amino acid sequences of plasmids for nanobody against (A) EC5026 and (B) TPPU. (C) The binding affinity of clones EC02, EC03, EC04, EC06 and EC09 to different concentrations of EC5026. (D) The binding affinity of clones TP01, TP02, TP03, TP04 to different concentrations of TPPU. Low absorbance means that the clone shows high binding affinity to the analyte.


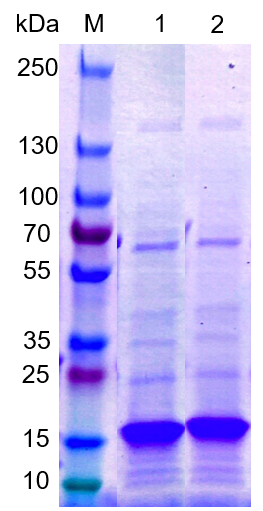


**Fig. S2** Characterization of nanobodies against sEH inhibitors by SDS–PAGE. Lane M, Marker. Lane 1, purified nanobody against EC5026 (Nb-EC03). Lane 2, purified nanobody against TPPU (Nb-TP02).

**
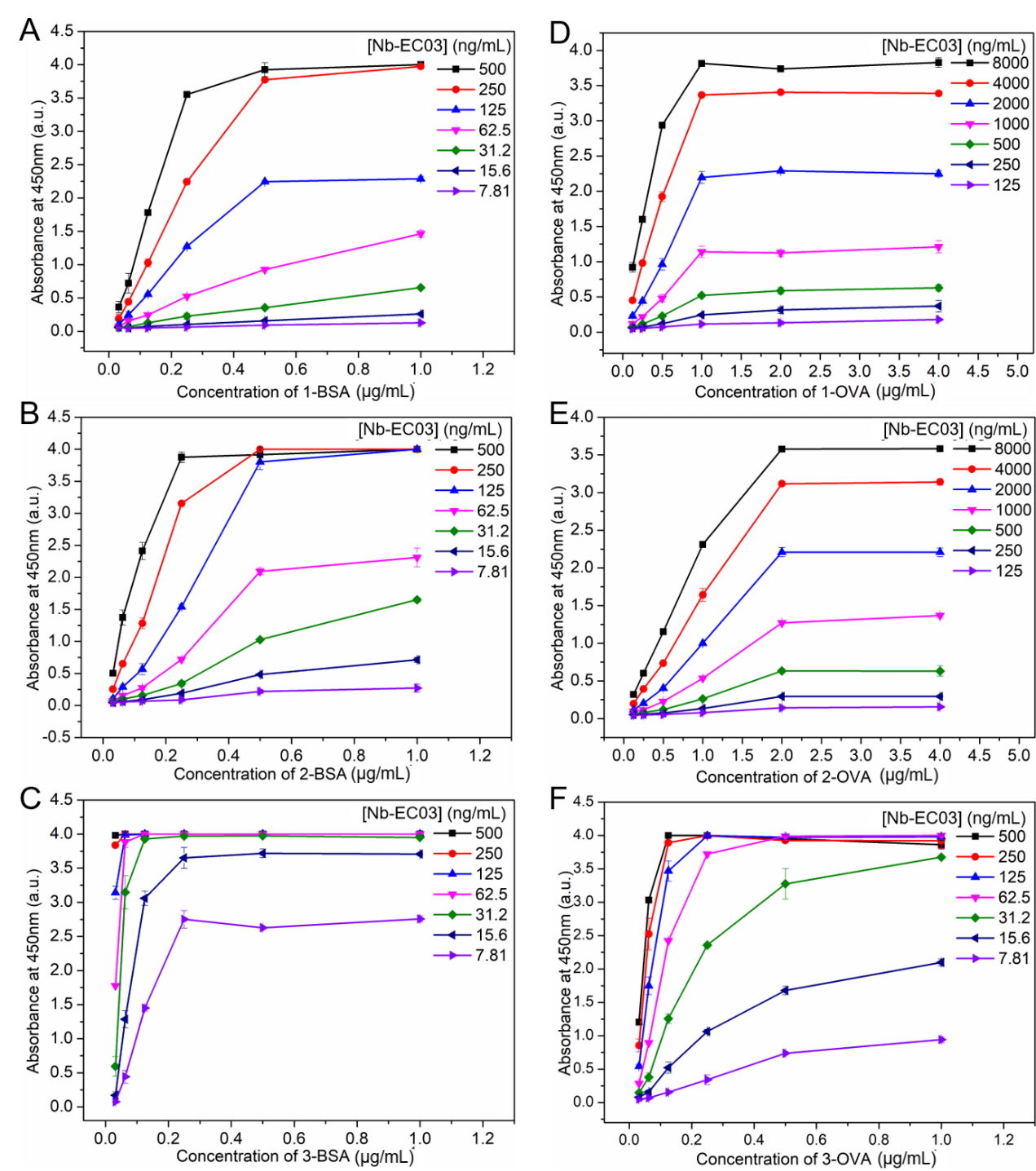
**

**Fig. S3** Optimization of concentrations of coating antigens and Nb-EC03 for the Nb-based ELISA with different coating antigens of (A) 1-BSA, (B) 2-BSA, (C) 3-BSA, (D) 1-OVA, (E) 2-OVA or (F) 3-OVA, respectively. The error bar represents the standard deviation (*n* = 3).

**
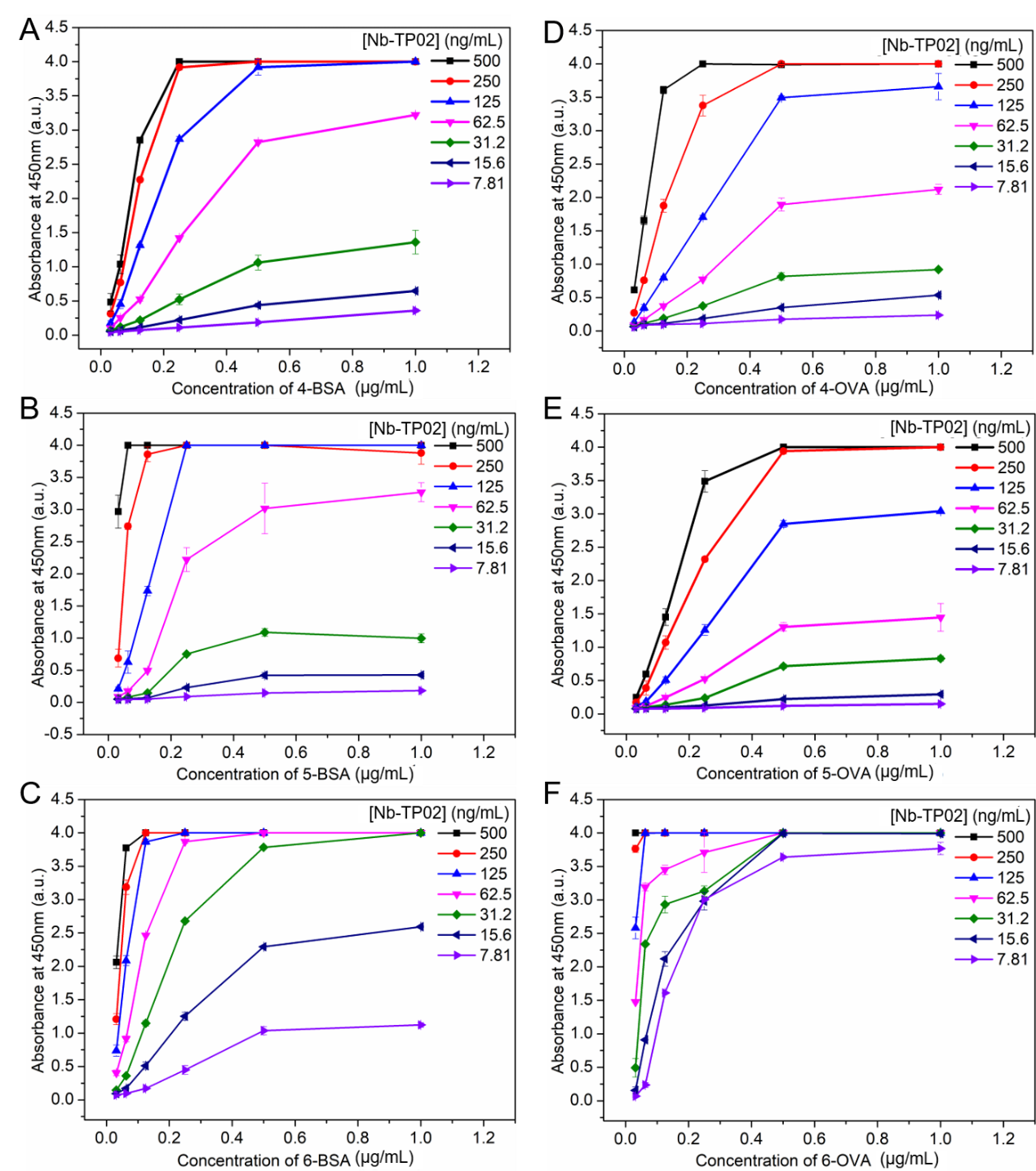
**

**Fig. S4** Optimization of concentrations of coating antigens and Nb-TP02 for the Nb-based ELISA with different coating antigens of (A) 4-BSA, (B) 5-BSA, (C) 6-BSA, (D) 4-OVA, (E) 5-OVA or (F) 6-OVA. The error bar represents the standard deviation (*n* = 3).


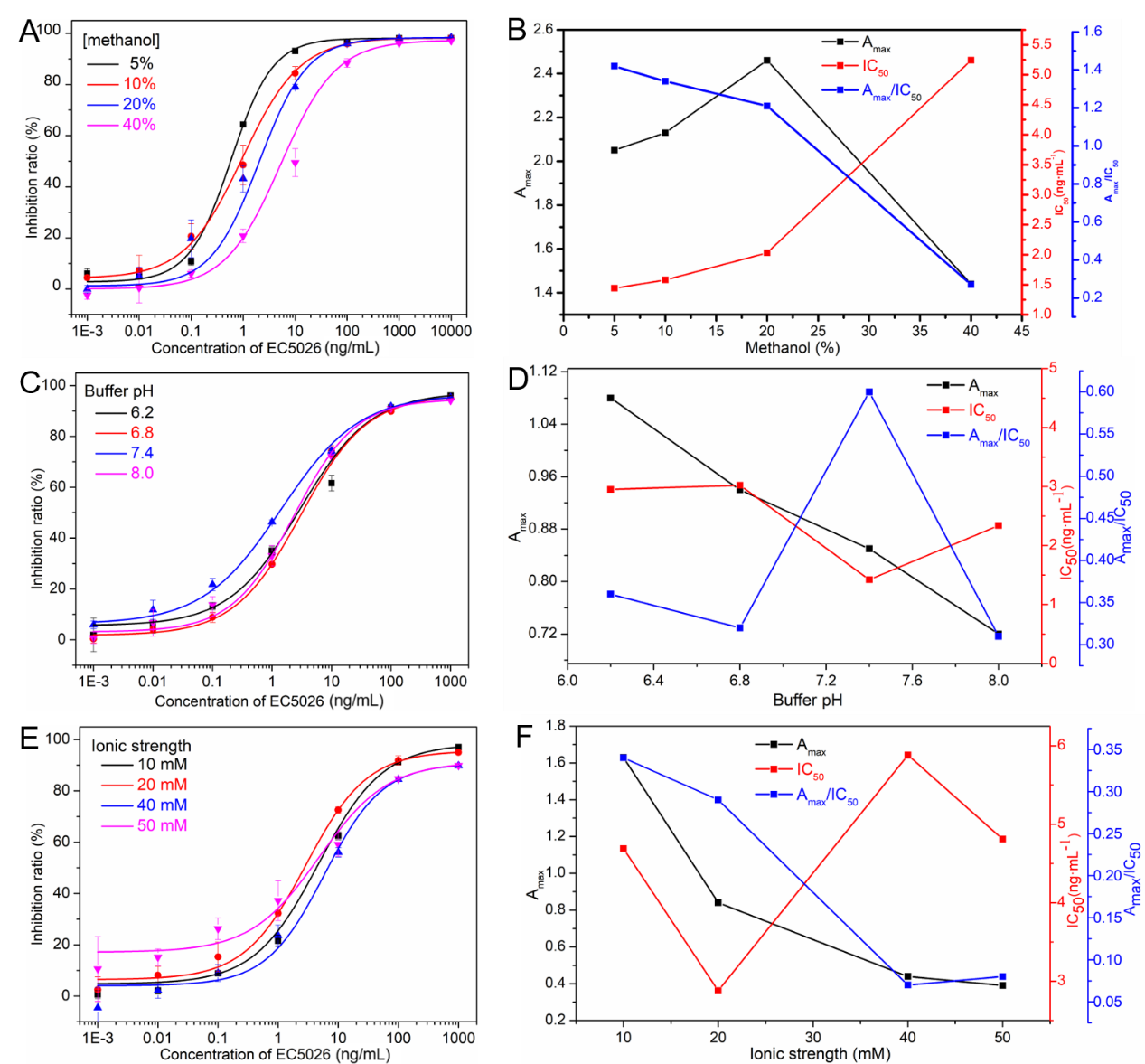


**Fig. S5** Standard curves of EC5026 with different physicochemical parameters and the relationship between three indicators *A_max_*, IC_50_ and *A_max_*/IC_50_ and physicochemical parameters of Nb-based ELISA for EC5026**.** (A) and (B) The concentration methanol. (C) and (D) pH values of buffer. (E) and (F) Ionic strength of buffer. Each point represents the average of triplicates for a given concentration (*n* = 3).


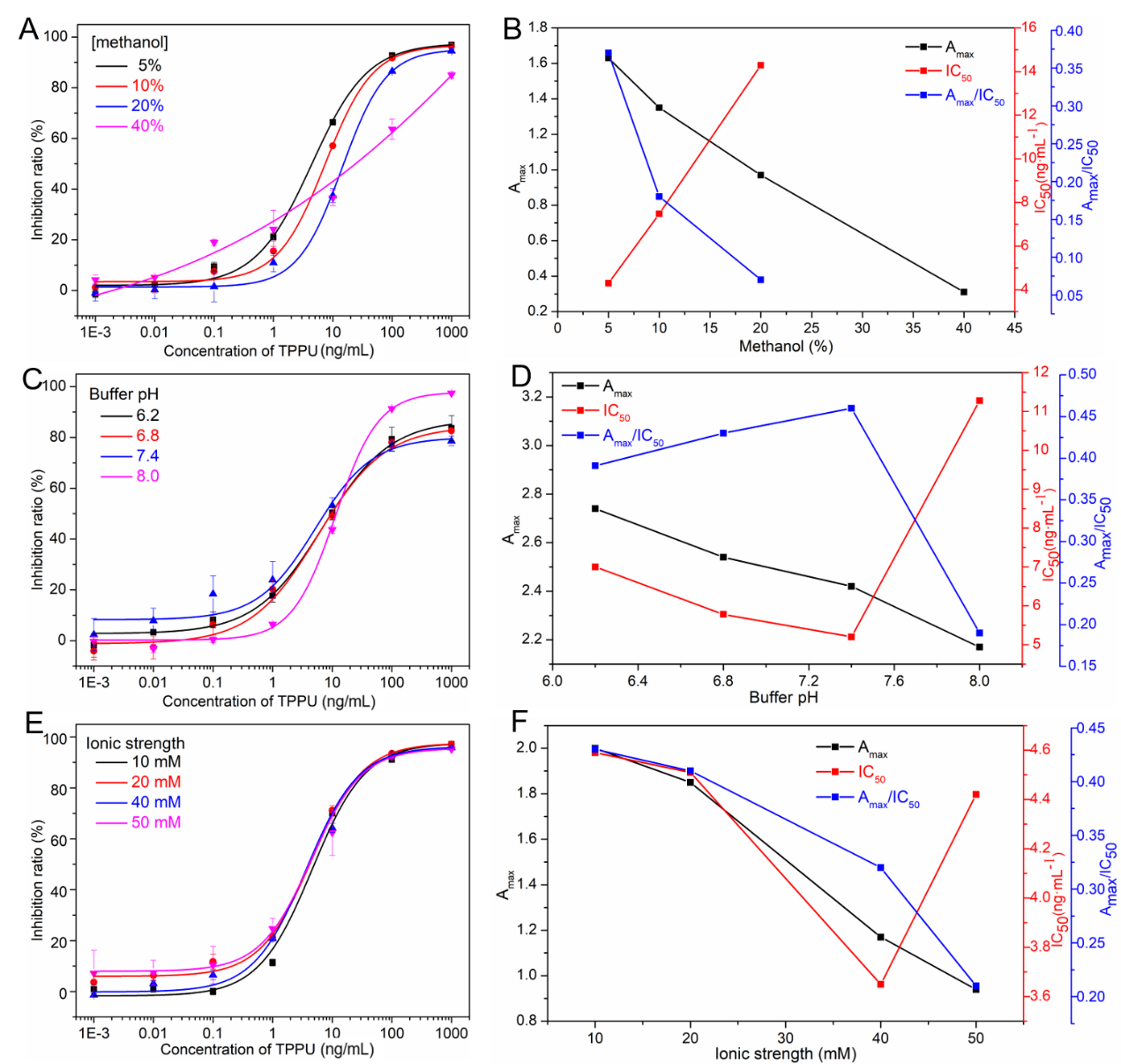


**Fig. S6** Standard curves of TPPU with different physicochemical parameters and the relationship between three indicators *A_max_*, IC_50_ and *A_max_*/IC_50_ and physicochemical parameters of Nb-based ELISA for TPPU**.** (A) and (B) The concentration methanol. (C) and (D) pH values of buffer. (E) and (F) Ionic strength of buffer. Each point represents the average of triplicates for a given concentration (*n* = 3).

**
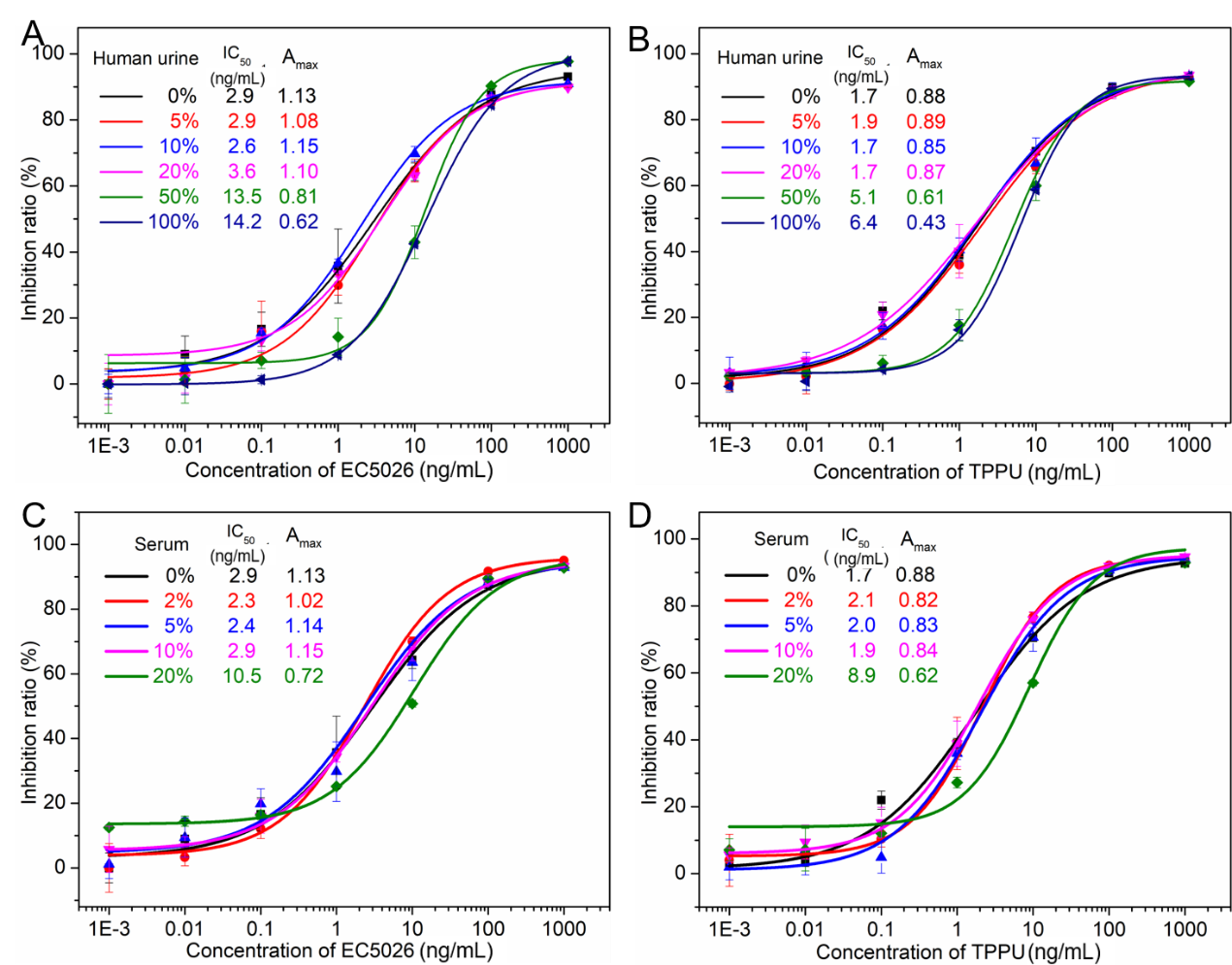
**

**Fig. S7** Standard curves for EC5026 in varying concentrations of (A) human urine and (C) fetal bovine serum. Calibration curves for TPPU in varying concentrations of (B) human urine and (D) fetal bovine serum. The error bar represents the standard deviation (*n* = 3).

**Table S1** Panning conditions

| Time of panning | Concentration of coating antigen (μg/mL) | EC5026 or TPPU  (μg/mL) | Times of Wash |
| --- | --- | --- | --- |
| R1 | 10 | 100 | 10 |
| R2 | 1 | 10 | 15 |
| R3 | 0.1 | 1 | 20 |

**Table S2** Optimization results of the concentration of coating antigens and nanobodies

| Analyte | Coating antigens | Optimal  [cAg] (μg/mL) | Optimal  [Nb] (ng/mL) | Absorbance^a^ |
| --- | --- | --- | --- | --- |
| EC5026 | 1-BSA | 0.5 | 250 | < 0.1 |
|  | 2-BSA | 0.5 | 125 | 0.2 |
|  | 3-BSA | 0.25 | 15.6 | 2.7 |
|  | 1-OVA | 1.0 | 2000 | < 0.1 |
|  | 2-OVA | 2.0 | 2000 | < 0.1 |
|  | 3-OVA | 0.5 | 31.2 | 0.8 |
| TPPU | 4-BSA | 0.5 | 62.5 | 0.1 |
|  | 5-BSA | 0.5 | 62.5 | 0.1 |
|  | 6-BSA | 0.25 | 15.6 | 3.5 |
|  | 4-OVA | 0.5 | 62.5 | 0.1 |
|  | 5-OVA | 0.5 | 125 | 0.1 |
|  | 6-OVA | 0.5 | 7.8 | 1.0 |

*^a^*The absorbance of the checkerboard ELISA when the concentration of nanobodies is 7.8 ng/mL

**Table S3** Nb-EC03 based ELISA results for homologous and heterologous coating antigens

| Immunogens for EC5026 | cAg  for panning | cAg for Nb-based ELISA | Curve parameters | | | |
| --- | --- | --- | --- | --- | --- | --- |
|  |  |  | Maximum absorbance | Slope | IC_50_  (ng/mL) | Minimum absorbance |
| 1-Thy  2-Thy | 1-BSA | 1-BSA*^a^* | 1.15 | 0.75 | 7.32 | 0.04 |
|  |  | 2-BSA*^a^* | 0.83 | 0.63 | 1.44 | 0.04 |
|  |  | 3-BSA*^b^* | 0.76 | 0.72 | 11.45 | 0.04 |
|  |  | 1-OVA*^a^* | 0.78 | 1.04 | 5.68 | 0.05 |
|  |  | 2-OVA*^a^* | 1.14 | 0.91 | 1.48 | 0.04 |
|  |  | 3-OVA*^b^* | 1.23 | 1.19 | 12.49 | 0.04 |

*^a^*Homologous coating antigens (hapten same as immunogens), *^b^*Heterologous coating antigens (hapten different from immunogens).

**Table S4** Nb-TP02 based ELISA results for homologous and heterologous coating antigens

| Immunogens for TPPU | cAg  for panning | cAg for Nb-based ELISA | Curve parameters | | | |
| --- | --- | --- | --- | --- | --- | --- |
|  |  |  | Maximum absorbance | Slope | IC_50_  (ng/mL) | Minimum absorbance |
| 4-Thy  5-Thy | 4-BSA | 4-BSA*^a^* | 1.91 | 1.02 | 2.53 | 0.04 |
|  |  | 5-BSA*^a^* | 1.20 | 1.10 | 4.53 | 0.04 |
|  |  | 6-BSA*^b^* | 1.24 | 1.17 | 2.40 | 0.04 |
|  |  | 4-OVA*^a^* | 0.73 | 0.98 | 2.76 | 0.04 |
|  |  | 5-OVA*^a^* | 0.76 | 0.88 | 2.44 | 0.04 |
|  |  | 6-OVA*^b^* | 1.04 | 0.99 | 2.08 | 0.04 |

*^a^*Homologous coating antigens (hapten same as immunogens), *^b^*Heterologous coating antigens (hapten different from immunogens).
